# Evaluation of Pulse Oximetry Alarm Fatigue and the Impact of SpO_2_ Thresholds on Clinical Workflow: A Prospective Observational Study in a Kenyan Neonatal Unit

**DOI:** 10.1177/30502225261427880

**Published:** 2026-03-16

**Authors:** Bazil M. Masabo, Augustine W. Waswa, Jesse Coleman, Morris Ogero, Grace Irimu, Amy Sarah Ginsburg, Dorothy Chomba, Millicent Parsimei, Cynthia Shitote, Ferdinand Okwaro, June K. Madete, William M. Macharia, J. Mark Ansermino

**Affiliations:** 1 Kenyatta University, Nairobi, Nairobi County, Kenya; 2 University of British Columbia, Vancouver, BC, Canada; 3 London School of Hygiene and Tropical Medicine, England, UK; 4 University of Nairobi, Nairobi County, Kenya; 5 University of Washington, Seattle, WA, USA; 6 Agha Khan University Nairobi, Nairobi County, Kenya

**Keywords:** pulse oximetry, alarm fatigue, neonatal care, clinical workflow, SpO2 thresholds

## Abstract

**Introduction:**

Effective pulse oximetry monitoring in a newborn unit requires clinician action. However, if thresholds are not adapted to each neonate, frequent alarms cause alarm fatigue, which impacts the quality of patient monitoring, staff workload and clinical workflow.

**Methods:**

We conducted an observational study from October 2021 to December 2022 in a Kenyan newborn unit which enrolled neonates through convenience sampling. Data were analysed in R using default thresholds of 85% to 96% for SpO_2_ and 90 to 200 bpm for PR.

**Results:**

Among the 49 neonates enrolled, median hourly alarms per patient were 12 visual and 9 audible. Half of SpO_2_ values were outside the set thresholds. The hourly alarm density per neonate was 4 SpO_2_ alarms and 1 PR alarm.

**Conclusions:**

The selected SpO_2_ alarm thresholds resulted in a high alarm burden which necessitates neonate-specific thresholds and delays. This improves monitoring specificity and sensitivity, enhancing neonatal care and effectiveness in resource-limited settings.

## Introduction

Preterm birth, defined as live birth before 37 weeks of gestation, is among the leading causes of neonatal mortality and is associated with long-term physical, neurodevelopmental, and socioeconomic effects.^1^ In 2020, an estimated 13.4 million preterm births were recorded worldwide, which is more than 1 in 10 infants. Despite being a global issue, there are regional disparities in the survival rates of preterm infants. Over 90% of preterm infants born in low-income countries, where most preterm births occur, die within the first few days of life.^2^ The death rate is less than 10% in high-income countries. In Kenya, the neonatal mortality rate is 21 deaths per 1000 live births, with preterm birth being the leading cause of death.

Preterm-born children are susceptible to adaptation problems in the early neonatal period and to poor outcomes due to the immaturity of the respiratory, cardiovascular, and especially cerebrovascular systems.^3^ Compared to term infants, preterm infants are 4 times more likely to die in the first 28 days of life, with mortality rates increasing proportionally with decreasing gestational age (GA) or birth weight.^4^ Extremely preterm infants frequently require some form of respiratory assistance to facilitate cardiopulmonary transition in the first hours of life.^5^ Respiratory distress syndrome (RDS) affects about 80% of neonates born at 28 weeks GA, and this percentage increases to 90% at 24 weeks GA.

The vulnerability of preterm infants typically necessitates critical, individualized, and continuous care in a neonatal intensive care unit (NICU) that can provide access to appropriate medical technologies. NICU medical technologies can enable lifesaving care through continuous monitoring of pulse rate (PR), oxygen saturation (SpO_2_), electrocardiography, and brain activity.^6^

The high number of physiological parameters requiring monitoring in the NICU and an expanding array of medical technologies have caused an increased frequency and volume of medical device alarms. Research shows that alarm fatigue, defined as a condition of sensory overload for healthcare providers (HCPs) exposed to an excessive number of alarms,^7^ is a serious problem in NICUs that can impact the quality of care and patient outcomes.^8^ Alarm fatigue can contribute to negative perceptions among HCPs, which can impede the delivery and quality of care and lead to an increased risk for adverse patient outcomes.^9,10^ Given that over 70% of clinical alarms do not require clinical intervention,^8^ overburdened clinicians can become desensitized to medical device alarms and subsequently become less responsive to clinically significant alarms.

There is growing interest in understanding and appreciating the importance of addressing alarm hygiene and how it contributes to alarm fatigue.^11^ Although medical device alarms are crucial for patient safety and augment clinical care, minimal evidence-based strategies exist to improve alarm hygiene. Different approaches have been tried, including an educational package on alarm management (the number of alarms, response to alarms, and appropriateness of settings) that resulted in a significant decrease in the number of unnecessary alarms and an improvement in the number of times where appropriate alarm settings were used.^12^ Other interventions include the adjustments of alarm thresholds and delay settings based on the patient’s condition.^13^ An evidence-based approach that balances patient safety with alarm sensitivity and specificity is thus critical for effective monitoring of patients.

In an effort to provide evidence to improve alarm fatigue for more effective management of neonates receiving continuous monitoring, we conducted a secondary analysis of alarm data obtained from pulse oximeters used in monitoring preterm neonates in a clinical feasibility study focused on using caffeine citrate to manage apnea of prematurity (AOP) in a single facility tertiary-care newborn unit (NBU) in Nairobi, Kenya.^14^

The aim of this study was to evaluate the frequency and clinical relevance of pulse oximetry alarms in a newborn unit, in a tertiary facility in Kenyan to determine how standardized SpO_2_ and pulse rate thresholds contribute to alarm fatigue among healthcare providers.

## Methods

### Study Design

This study is part of a larger quality improvement study which included administration of caffeine citrate and continuous monitoring for neonates at risk of AOP that was conducted between October 2021 and December 2022. The study included a 4-month formative research phase followed by the development and implementation of an apnea of prematurity (AOP) clinical-care bundle prototype. Implementation and improvement of the clinical-care-bundle prototype were done in the second phase. The baseline pulse oximetry data was used to provide contextualized insights on care practices within the NBU to inform the development of a context-sensitive AOP prototype clinical-care bundle.

Recognized as a reference site for newborn care standards in Kenya, this NBU in a tertiary teaching and referral healthcare facility in Nairobi, Kenya, has considerable potential to influence clinical practices more widely. The NBU staff comprised of 5 neonatologists, 7 neonatology fellows, 1 pediatrician, 3 medical officers, and 12 to 15 pediatric residents. One medical officer and 1 resident provided night coverage. The NBU had a complement of 80 nurses working in shifts, with an average of 13 on each shift. Training sessions on the management of AOP were conducted for all staff, including nurses, residents, medical officers, consultants, and nutritionists.

Typically, 150 neonates are admitted monthly to the NBU, which has an average bed capacity of 60 neonates. Neonates often share essential equipment such as cots, radiant warmers, and incubators. Annually, 13% of NBU admissions are very low birth weight neonates (1000-1499 g), with 85% admitted on the day of birth (Source: Hospital NBU Database). For this study, a convenience sampling of consecutive neonates admitted to the NBU during the defined study period who were born less than 34 weeks gestational age (GA), or with a birth weight less than 1500 g where the GA was unknown, provided the estimated GA was less than 34 weeks as determined using New Ballard scoring, was screened for enrollment. Neonates receiving caffeine citrate or aminophylline for AOP were enrolled subject to their caregivers providing written informed consent for data use. Routine continuous pulse oximetry was implemented during the study, and data from the devices were captured and analyzed.

### Study Procedures

Rad-G™ pulse oximeters with reusable Masimo Yi sensors were used for continuous monitoring. Formal training in using the pulse oximeters was provided to all nursing staff. The devices were connected to the neonates by the NBU nurses as soon as the neonates were admitted to the NBU. Routine clinical hygiene measures were followed; including strict hand hygiene during attachment and detachment of the sensors and cleaning the pulse oximeters and sensors when transferring from one neonate to another. The study protocol recommended that the location on the neonate’s body where the pulse oximeter sensor was attached should be changed every 3 hours, but this was not consistently implemented.

Based on clinical consensus, default alarm thresholds were set during device setup (see Table 1). These settings could be changed when the neonate was off supplemental oxygen unless needed for other clinical indications. The study protocol recommended that the upper threshold be disabled for neonates not on supplemental oxygen, but this was not consistently followed.

**Table 1.** Default Alarm Thresholds.

Parameter	Threshold
Sensitivity	Normal
Averaging time	8 seconds
Rapid desaturation	10%
Alarm delay	15 seconds
Fast sat	OFF
SpO_2_	Upper threshold: 96% Lower threshold: 85%
PR	Upper threshold: 200 bpm Lower threshold: 90 bpm

Based on the device identifiers, the study nurses recorded which neonate was attached to a specific device each day. At the end of each day, data were transferred from each device to a universal serial bus (USB) drive and then to a laptop computer running the Masimo Trace Software^@^. Data at 0.5 hertz (Hz) resolution were exported into comma-separated values (CSV) files for further analysis.

### Patient and Public Involvement

While patients and the public were not involved in the study design, caregivers provided written informed consent for the use of clinical data.

### Data and Statistical Analysis

Data at 0.5 Hz resolution were used. Missing values were replaced with nulls, and invalid entries for SpO_2_ and PR were removed to ensure data integrity. R Studio 4.2.3 and dplyr, tidyverse, ggplot, gridExtra, lubridate packages were used to clean and analyze data and perform statistical analyses. Clinical events were split into six columns for each unique alarm entry category for detailed analysis. The description of relevant terms is captured below (see Table 2).

**Table 2.** Description of Device Terminologies.

Term	Description
Pulse oximeter alarm	An alert triggered by the pulse oximeter when a measured value falls outside a set range
Audible alarm	An audible alert sounded by the pulse oximeter
Visual alarm	A visual alert displayed by the pulse oximeter
System alarm	An audible and/or visual alarm due to a system event, such as a sensor being detached or motion detected
Clinical alarm	An audible and/or visual alarm due to any out-of-range parameter
SpO_2_	Percentage of oxygenated blood measured with pulse oximetry
Pulse rate (PR)	Number of heartbeats per minute measured with pulse oximetry
Perfusion index (PI)	Pulse oximetry-specific ratio of pulsatile volume of blood flow compared to a baseline measurement; used as a surrogate for peripheral perfusion to guide appropriate sensor placement

We focused our analysis on SpO_2_ and PR, utilizing R’s quantitative tools and visualizations (histograms, bar graphs, pie charts). In addition, we excluded rows with invalid entries for SpO_2_ (“Invalid functional SpO_2_”) and PR (“Invalid PR”), together with alarm entries due to low SpO_2_ signal identification and quality indicator (SIQ).

We quantified the frequency and duration of visual, audible or audio-visual alarms based on a 2 second data sampling rate. We defined an alarm event as a continuous sequence of alarm triggers. To avoid overcounting, we grouped consecutive triggers together on condition that a new alarm was only recorded if the alarm type changed or if there was a gap of more than 30 seconds between the occurrences. Total alarm duration was then calculated by multiplying the number of consecutive 2 second data cells by 2.

### Ethical Approval and Informed Consent

This study received approval from Kenyatta National Hospital/University of Nairobi Ethics Review Committee (P922/11/2021) and the Aga Khan University Nairobi Ethics Committee 2019/IERC 165 (V1), Nairobi, Kenya (Supplemental File 5).

All the stakeholders involved in the study provided written informed consent. No data were retained for caregivers who declined their participation in the study. In addition, administration of quality care to the neonates whose parents declined to consent was assured to the participants and no participant was given special treatment regardless of his or her choice to participate in the study.

Participants were allowed to sign non-disclosure agreement within the informed consent to maintain the confidentiality of the information given. All the data were stored in laptops and secured in the lockers at Aga Khan University, Nairobi. This was to safeguard all the study information and data from non-study team members.

## Results

### Socio-Demographic and Clinical Characteristics

The study included neonates born at less than 34 weeks GA or with a birth weight less than 1500 g if GA was unknown. Of the 49 neonates enrolled in the study, there were more males (31; 63%) than females (18; 37%), and the median birth weight at admission was 1.3 kg (IQR, 329 g). Seven (14%) neonates died during the study. Respiratory support was provided during 28% of patient days, including intubation and mechanical ventilation during 194 (13%) days and continuous positive airway pressure (CPAP) during 219 (15%) days.

We recorded 16 212 hours of monitoring data overall. Continuous monitoring durations of SpO_2_ and PR varied across neonates from a minimum of 16 hours up to a maximum of 41 days, with a median monitoring duration of 11.2 (IQR, 14.2) days. We excluded 9.97% of the total duration of monitoring data for poor data quality, mainly due to system events, including sensor detachment, low perfusion index, and cable disconnection. These system events frequently resulted in non-values (74.8% for SpO_2_ and 73.6% for PR). The poor-quality data contained 31 058 (5.4%) alarms in total.

### Overall Alarm Burden

There were 565 413 clinical alarms during monitoring, with 87% (493 577) lasting 30 seconds or less. Audible alarms accounted for 44% of the total alarm burden and had a median duration of 8 (IQR, 36) seconds. The alarms over 30 seconds long lasted a median duration of 78 (IQR, 124) seconds. Audible alarms lasted longer than visual alarms, and almost all alarms that lasted more than 2 minutes were audible. Alarms of durations more than 5 minutes and 10 minutes accounted for 4.2% and 2.1% of audible alarms, respectively.

**Figure 1.**
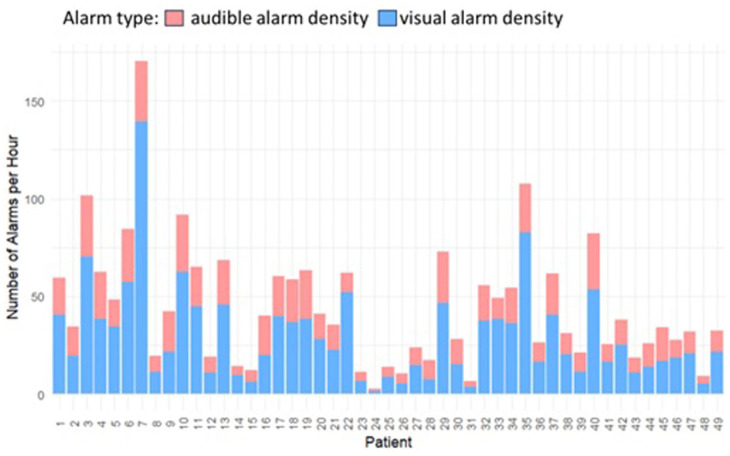
Median alarm density per neonate comparing visual versus audible alarms.

Each neonate experienced a median of 9993 (IQR, 15 600) alarms throughout monitoring, translating to a median duration of 97 (IQR, 123) alarm hours. The highest hourly count was 201 alarms, and the most alarm-burdened day had 2179 alarms. The median alarm density (number of alarms per hour) per neonate was higher for visual (12 [IQR, 40]) than for audible (9 [IQR, 21]) alarms (Figure 1).

### Oxygen Saturation Alarms

**Figure 2.**
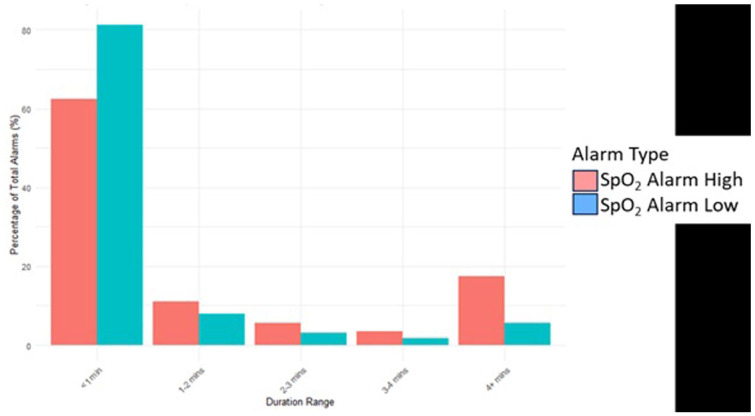
Percentage of SpO_2_ alarms with different durations as a percentage of total SpO_2_ alarms.

**Figure 3.**
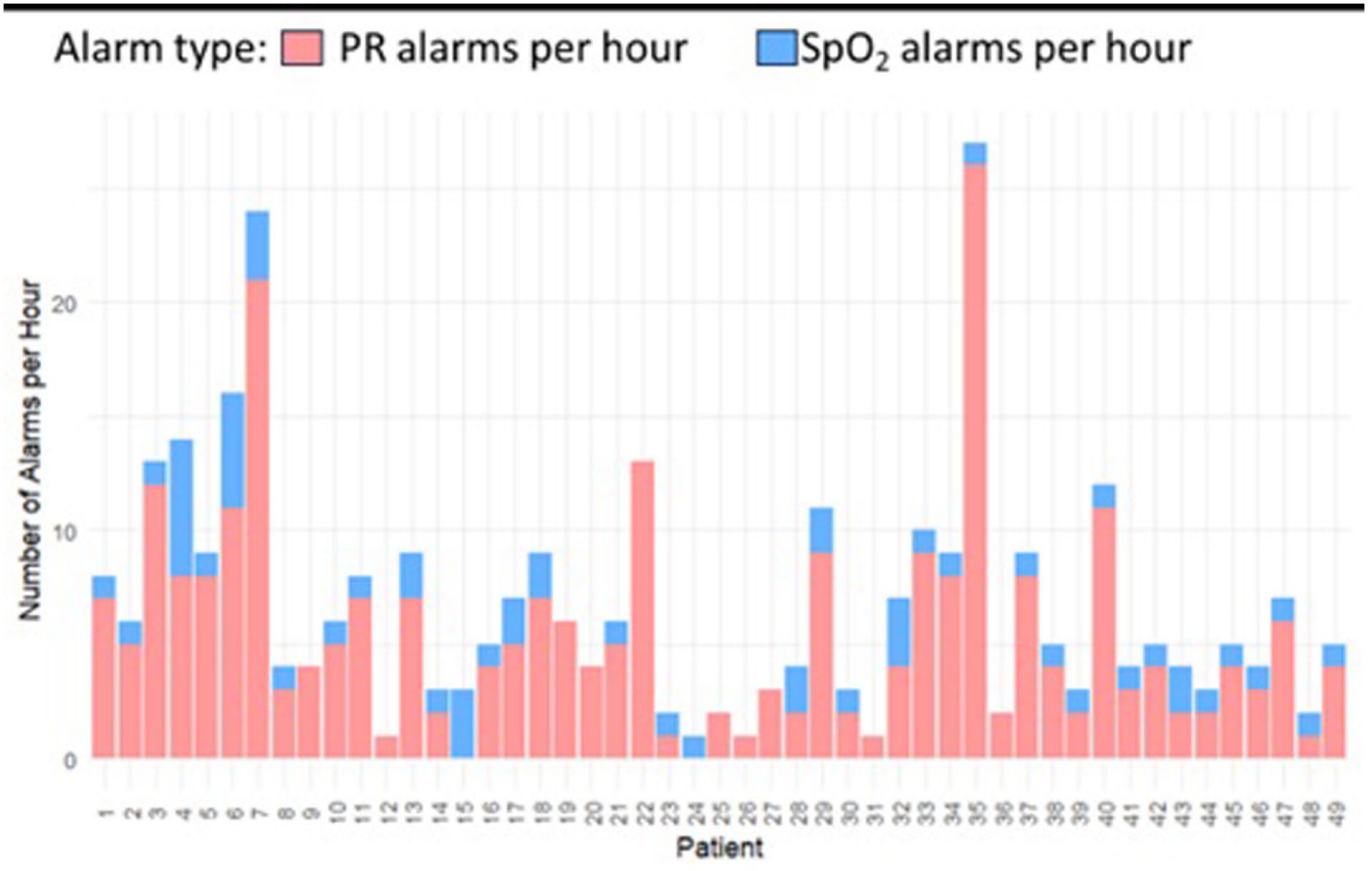
Comparison of SpO_2_ alarm frequency versus PR alarm frequency across the study population.

With an overall median SpO_2_ value of 96% (IQR, 5%), we recorded 98 047 SpO_2_ alarms, 56 312 (57.4%) high, and 41 735 (42.6%) low alarms. Of the 50% recorded SpO_2_ values that were outside the set threshold range, 44.5% were greater than the upper threshold of 96% (of these, 36% with 100% SpO_2_; 20% with 99% SpO_2_; 22% with 98% SpO_2_; and 22% with 97% SpO_2_). The median duration of SpO_2_ alarms was 20 (IQR, 72) seconds. Figure 2 shows that most alarms − 80% of low alarms and 60% of high alarms − were less than a minute long, while longer duration (above 4 minutes) alarms were predominantly high SpO_2_ alarms (over 15%). Overall, 5.78% of the SpO_2_ values were below 85%, leading to a median of 1.7 (IQR, 0.6) alarms per hour per neonate. On average, each neonate had a median of 1643 (IQR, 2950) SpO_2_ alarms, with the highest number being 13 680 alarms. The median SpO_2_ alarm burden for both low and high alarms was 4 (IQR, 5) alarms per hour per neonate. Figure 3 shows a higher alarm density of SpO_2_ alarms than PR alarms.

### Pulse Rate Alarms

**Figure 4.**
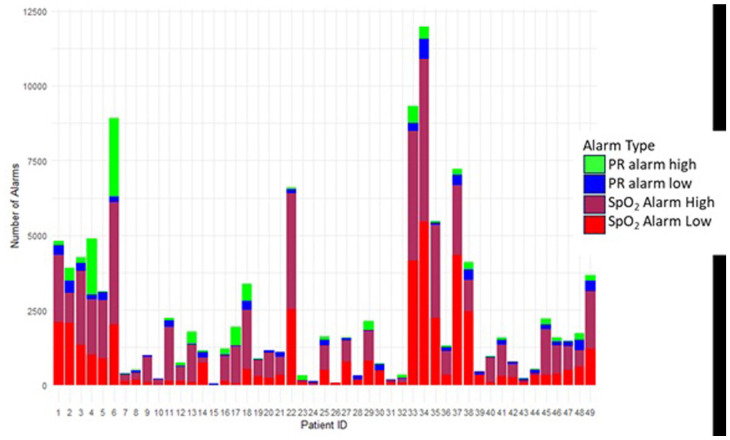
Distribution of the medical alarms per patient categorized by alarm type.

With a mean (standard deviation [SD]) PR value of 158 (19.87) beats per minute (bpm) throughout monitoring, we recorded 18 218 PR alarms, 58.5% high PR and 41.5% low PR alarms. The mean (SD) alarm density was 1 (1.17) PR alarm per hour per neonate, with 2% of the data lying outside the thresholds. The median number of alarms per neonate was 199 (IQR, 347), and each neonate experienced, on average, 371 PR alarms throughout monitoring with a median of 71 (IQR, 156) alarms per day per neonate. PR alarms contributed a smaller portion of the total alarm burden than SpO_2_ alarms (Figure 4). When monitoring PR, 14 096 alarms (77%) lasted shorter than a minute. High alarms were the majority, both for alarms with durations below a minute and for longer-duration alarms. The median duration for PR alarms was 18 (IQR, 44) seconds.

## Discussion

This study provides a comprehensive analysis of pulse oximeter alarm data in a resource-limited newborn unit. Our primary finding reveals a staggering alarm burden. Out of the total 565 413 alarms recorded, 87% lasted 30 seconds or less. This high frequency of short-duration alarms suggests that many alerts do not represent sustained clinical deterioration, hence are unwarranted. Over 85% of alarms lasted 1 minute or less, with many lasting only 2 seconds. It is assumed that multiple small changes in SpO_2_ and PR were responsible for many of the alarms, and not all were likely indicative of clinically significant events. Since neonates are prone to high alarm burdens, transient changes in SpO_2_ and PR could be due to intrinsic neonatal physiological fluctuations, patient movements, sensor interference, and poor signal quality. Many alarms were self-resolving without clinical intervention, thereby contributing to a large proportion of non-actionable alarms.

Frequent non-critical alarms that are clinically irrelevant can create habituation among HCPs, leading to alarm fatigue and reduced responsiveness to real clinical events.^15^ This excessive alarm rate is a primary driver of “alarm fatigue.” When healthcare providers are bombarded by non-actionable alerts, it leads to sensory overload and provider desensitization. This reduces the likelihood of prompt responses to true emergencies, as critical signals are lost in the constant noise. Persistent exposure to non-actionable alarms can result in slower response times and missed or delayed interventions during emergencies, with serious and sometimes deadly consequences.^16^

Furthermore, the frequent arousal of neonates may have deleterious effects on infant growth, development, and health. Sleep is critical for physical growth, brain maturation, and autonomic regulation in neonates. Frequent awakenings due to alarms have been linked to increased cortisol levels, indicating heightened stress, which can interfere with immune function and neurodevelopmental progress. Frequent and unnecessary disruption of sleep cycles caused by audible alarms may have long-term developmental implications for neonates.^10^

## Alarm Hygiene

### Introducing Alarm Hygiene

Alarm hygiene is a set of strategies and practices implemented to manage and reduce the frequency of false alarms in systems, particularly in industrial and healthcare settings.^17^ Effective alarm hygiene ensures that alarms are meaningful and actionable and improve safety and efficiency. There is an intricate balance between sensitivity and specificity to achieve a high signal-to-noise ratio and avoid missed events without unnecessary alarms.

When proper alarm hygiene is not observed, clinicians are exposed to a high number of alarms, which leads to alarm fatigue. The consequence is alarm desensitization, leading to missed alarms and delayed interventions.

### Increasing Alarm Delay

With 87% (493 577) of alarms in our study lasting 30 seconds or less, increasing the alarm delay is one effective method to reduce the alarm burden. A delay in the time taken for an alarm to trigger allows transient changes in the patient parameter to self-correct. This can filter out non-actionable alarms due to brief fluctuations in the measured parameter or interferences from patient movements. Our findings are consistent with existing literature on alarm management. Notably, Welch et al demonstrated that implementing a modest 15-second alarm delay can reduce total alarm frequency by 70%. Furthermore, lowering alarm limits to 88% alongside this delay can reduce the total burden by over 85% while still preserving the detection of actionable events. Alarm delays allow improvement in alarm specificity without affecting a reasonable clinical response time.^18^ Moreover, modifications of alarm delays to suit patient conditions could further reduce the overall number of alarms in clinical environments,^19^ particularly for high-frequency alarms like low SpO_2_ and SpO_2_ probe off.

We observed in our study that of the alarms lasting less than 30 seconds, the greatest proportion lasted less than 10 seconds. This indicates that many alarms may not be clinically significant but could still contribute to alarm fatigue. Therefore, implementing a 10-second delay as a filter could mitigate the issue by reducing transient, momentary alarms that often do not reflect critical patient events.

### Modifying Oxygen Saturation Alarm Thresholds

Modifying oxygen saturation alarm thresholds according to patient conditions and treatments presents another technique for reducing the frequency of non-actionable alarms. A 2% change in the lower SpO_2_ threshold could reduce the alarm burden by up to 50%.^19^ The results suggest that optimizing SpO_2_ thresholds may further improve the specificity of alarms, minimize alarm fatigue, and enhance overall patient monitoring efficiency.^20^

The SpO_2_ alarm thresholds used in this study captured a substantial proportion of data outside the thresholds, with the overwhelming majority exceeding 95%, the upper threshold, and 38% due to SpO_2_ values of 100%. For premature neonates on supplemental oxygen, a high SpO_2_ value indicates excessive oxygen administration, which can increase the risk of retinopathy. However, for neonates on room air, a high SpO_2_ value would be clinically reassuring. Thus, we recommend a default upper threshold of 95% for those on supplemental oxygen and no upper threshold for those on room air. These changes, combined with a standardized 15-second delay, would significantly reduce unnecessary noise while focusing provider attention on neonates at the highest clinical risk.

Reducing the low SpO_2_ alarm threshold from 90% to 85% can decrease alarms by 75%.^13^ Less than 6% of alarms in our study were for SpO_2_ values below 85%, the lower threshold. Adjusting the lower threshold to 80% could significantly reduce the proportion of excluded data. A theorized minor adjustment (eg, 80.2%) had a pronounced impact, excluding only 3.4% of the data compared to 6% at 85%. This suggests a high sensitivity of data distribution around the lower threshold. Clinically, this indicates the importance of selecting alarm thresholds that balance sensitivity (capturing relevant hypoxemia events) and specificity (avoiding unnecessary alarms that may not represent critical events). We recommend a lower threshold of 80%,^21^ but with individualized threshold adjustment for infants with SpO_2_ values consistently below this threshold despite context-appropriate interventions.

### Modifying Pulse Rate Alarm Thresholds

Complementary to the low SpO_2_ alarm, the low PR alarm reflects the neonate’s physiological response to hypoxemia and could be used to escalate the urgency of the SpO_2_ alarm. A significant drop in SpO_2_ initiates a series of compensatory mechanisms to ensure adequate organ oxygenation. Increased cardiac output is initially common, but in severe cases, a bradycardic response takes over as the body’s final attempt to reduce oxygen consumption. In our study, the PR thresholds of 90 to 200 bpm resulted in 2% of the data being outside the thresholds. For the low PR threshold of 90 bpm, only 0.92% of the data fell below the threshold compared to 1.1% at a theoretical threshold of 98%. We recommend a low PR threshold of 90 bpm.

### Combination of Both Methods

A combination of both optimizing alarm thresholds and utilizing alarm delays provides a higher reduction in the alarm burden, which can be critical in balancing the competing priorities of patient safety and alarm fatigue mitigation. Lowering alarm limits to 88% with a 15-second delay can reduce alarms by over 85%.^13^ These 2 settings offer significant alarm reduction while preserving actionable alarms and thus maintaining patient safety, which is a focus of alarm hygiene.

### Data-Driven Adjustments

These data-driven interventions could lead to daily alarm densities of less than 10 alarms per day even in busy settings.^22^ This reduction highlights how modest adjustments to alarm settings can meaningfully impact alarm frequency in busy clinical environments. Furthermore, analyses of several alarm burden reduction methodologies indicate that critical monitoring, such as during events leading to code blue or high-acuity transfers, is not compromised,^22^ by data-driven adjustments to alarm delays to suit patient conditions.

### Organizational Change

Optimizing and individualizing alarm thresholds and delays can reduce the alarm burden, but also should be combined with organizational change, using traditional quality improvement methodologies, to optimize responsiveness to alarms.^8^ Other strategies to reduce alarm burden include selecting appropriate technologies, such as sensors specifically designed for use with premature neonates, and optimizing signal quality by proper placement of sensors. Excluding external light, keeping the environment warm, preventing motion of the sensor and cable, and using algorithms to track the dynamic changes in monitoring, in addition to thresholds, are also considerations.

## Limitations

Our study was limited by the convenience sampling strategy, a small sample size, and a potential lack of generalizability to other patient populations and healthcare settings. In addition, in our analysis, we classified 9.97% of the data as poor quality, making up about 31 053 (5.4%) alarms. Common causes of poor-quality data were system events, including sensor detachment, low perfusion index, and cable disconnection. Alarms triggered by such events can contribute to the alarm burden. Since the study enrolled neonates who frequently moved their hands and feet, sensor detachments are expected to be common. Therefore, good practice for sensor placement is necessary.

## Conclusions

In the context of our study, these results reinforce the idea that optimizing alarm settings is an effective strategy for reducing alarm burden. Our findings also underscore the importance of data-driven adjustments, where thresholds are tailored based on clinical priorities and patient population needs. These data-driven adjustments ensure that critical events remain detectable while reducing the noise of non-actionable alarms.

Since premature neonates are prone to high alarm burdens due to their unique physiology and care needs, data-driven alarm management strategies are more critical in the NICU. Neonate-specific alarm thresholds and well-adjusted alarm delays can improve the specificity and sensitivity of monitoring devices. When device alarm settings are coupled with appropriate organizational adaptations, patient care and safety are enhanced while HCP exposure to excessive alarms is mitigated.

## Supplemental Material

sj-pdf-1-gph-10.1177_30502225261427880 – Supplemental material for Evaluation of Pulse Oximetry Alarm Fatigue and the Impact of SpO2 Thresholds on Clinical Workflow: A Prospective Observational Study in a Kenyan Neonatal Unit Supplemental material, sj-pdf-1-gph-10.1177_30502225261427880 for Evaluation of Pulse Oximetry Alarm Fatigue and the Impact of SpO2 Thresholds on Clinical Workflow: A Prospective Observational Study in a Kenyan Neonatal Unit by Bazil M. Masabo, Augustine W. Waswa, Jesse Coleman, Morris Ogero, Grace Irimu, Amy Sarah Ginsburg, Dorothy Chomba, Millicent Parsimei, Cynthia Shitote, Ferdinand Okwaro, June K. Madete, William M. Macharia and J. Mark Ansermino in Sage Open Pediatrics
