# Supplementary material for: Evaluation of Pulse Oximetry Alarm Fatigue and the Impact of SpO2 Thresholds on Clinical Workflow: A Prospective Observational Study in a Kenyan Neonatal Unit
Source: Sage Open Pediatr. 2026 Mar 16;13:30502225261427880. doi: 10.1177/30502225261427880 (PMC13010038; doi:10.1177/30502225261427880)
Supplement: sj-pdf-1-gph-10.1177_30502225261427880 – Supplemental material for Evaluation of Pulse Oximetry Alarm Fatigue and the Impact of SpO2 Thresholds on Clinical Workflow: A Prospective Observational Study in a Kenyan Neonatal Unit [file sj-pdf-1-gph-10.1177_30502225261427880.pdf]

Feasibility of management of apnea of prematurity with caffeine citrate at a tertiary health care facility in Kenya. A quality improvement study

A study of the Evaluation of Technologies for Neonates in Africa (ETNA) platform

Sponsor: Aga Khan University, Nairobi, Kenya

Version 1.4
February 09, 2023

Principal investigator: William M Macharia
Co-principal investigators: Grace Irimu, Amy Ginsburg
Co-investigators: Mary Waiyego, Mark Ansermino, Jesse Coleman, Ferdinand Okwaro, Jasmit Shah, Roseline Ochieng, Florence Murila
Protocol team: William Macharia, Grace Irimu, Amy Ginsburg, Ferdinand Okwaro, Jasmit Shah

Confidentiality statement
This document is confidential and is to be distributed for review only to investigators, potential investigators, consultants, ETNA study staff, applicable regulatory authorities, and applicable independent ethics committees or institutional review boards.

Evaluation of Technologies for Neonates in Africa (ETNA) project

Contents

ABBREVIATIONS AND ACRONYMS 4
INVESTIGATOR TEAM 5
PARTICIPATING INSTITUTIONS 6
PROTOCOL OUTLINE 6
1 STRUCTURED ABSTRACT 7
2 BACKGROUND AND RATIONALE 7
2.1 Study justification 9
3 STUDY HYPOTHESIS, OBJECTIVES, AND ENDPOINTS 9
3.1 Hypothesis 9
3.2 Objectives 9
4 METHODS 10
4.1 Study site 10
4.2 Study design 10
4.3 Study population 11
4.4 Sample size 11
4.5 PHASE I: FORMATIVE RESEARCH 12
4.5.1 Neonate screening 12
4.5.2 Informed consent process 12
4.5.3 Enrollment of neonates 13
4.5.4 Neonate data collection 13
4.5.5 Stakeholder qualitative data collection 14
4.5.6 Transition from formative research phase to quality improvement implementation phase 15
4.6 PHASE II. QUALITY IMPROVEMENT IMPLEMENTATION 16
4.6.1 Neonate screening, informed consent process, neonate enrollment, neonate data collection 17
5 TIMELINE 18
6 STUDY PROCEDURES 19
6.1 Qualitative evaluations 20
6.2 Withdrawal and early termination 20
6.3 Study termination 20
7 STUDY STAFF TRAINING REQUIREMENTS 20
8 DATA COLLECTION, MANAGEMENT, AND ANALYSIS 21
8.1 Data collection forms 21
8.2 Source documents 21
8.3 Data management and monitoring 21
8.4 Missing data 22
8.5 Data analysis 22
8.6 Qualitative data collection 22
8.7 Data access 22
8.8 Data storage 23
9 SAFETY MONITORING 23

10 ETHICAL CONSIDERATIONS 23
10.1 Principles for clinical research 23
10.2 Institutional review boards and independent ethics committees 23
10.3 Informed consent documentation 24
10.4 Study discontinuation 24
10.5 RISKS, BENEFITS, AND CONSTRAINTS 24
10.5.1 Risks to participants 24
10.5.2 Benefits to participants 25
10.5.3 Potential constraints 25
11 DISSEMINATION OF STUDY RESULTS 25
12 REFERENCES 26
13 APPENDICES 28
13.1 Appendix I. Schedule of Study Procedures and Evaluations 28
13.2 Appendix II. Informed Consent Forms 30
13.3 Appendix III. Study Data Elements 44

ABBREVIATIONS AND ACRONYMS

AKU	Aga Khan University, Nairobi
CI	confidence interval
CIN	Clinical Information Network
CPAP	continuous positive airway pressure
DCF	data collection form(s)
ERC	ethics review committee
ETNA	Evaluation of Technologies for Neonates in Africa
FGD	focus group discussion(s)
GCP	Good Clinical Practice
HCA	healthcare administrator
HCP	healthcare provider
ICF	informed consent form
IDI	in-depth interview(s)
IRB	institutional review board
MCPM	multiparameter continuous physiological monitoring
PDSA	plan-do-study-act
PI	principal investigator
PID	participant identification
REDCap	Research Electronic Data Capture
UoN	University of Nairobi
WHO	World Health Organization

Evaluation of Technologies for Neonates in Africa (ETNA) project

INVESTIGATOR TEAM

Principal investigator (PI):
William Macharia, MBChB, MMed, MSc
Pediatrician and Clinical Epidemiologist
Aga Khan University, Nairobi, Kenya

____________________ 09/02/2023__________
PI signature Date

Co-PIs
Grace Irimu, MBChB, Mmed, PhD
Pediatrician and Health Systems Researcher
University of Nairobi, Nairobi, Kenya

Amy Ginsburg, MD, MPH
Infectious Diseases Physician and Epidemiologist
University of Washington, United States

Co-investigators
Mary Waiyego, MBChB, MMed
Neonatologist
Kenyatta National Hospital, Nairobi, Kenya

Florence Murila, MBChB, MMed
Neonatologist
University of Nairobi

Roseline Ochieng, MBChB, MMed
Neonatologist
Aga Khan University, Nairobi

Jesse Coleman, PhD
Global Health Researcher
University of British Columbia, Canada

Ferdinard Okwaro, PhD
Medical Anthropologist
Aga Khan University, Nairobi

Jasmit Shah, PhD
Biostatistician/Bioinformatician/Data Scientist
Aga Khan University, Nairobi

Mark Ansermino, MBBCh, MMed, Msc (Info), FRCPC, FFA (SA)
Pediatric Anesthesiologist
University of British Columbia, Canada

PARTICIPATING INSTITUTIONS

**Study oversight, management, and operations**	Aga Khan University, Nairobi, Kenya
**Collaborators**	Kenyatta National Hospital, Nairobi, Kenya<br>University of Washington, Seattle, WA, USA
**Funding**	Bill & Melinda Gates Foundation, Seattle, WA, USA
**Study site**	Kenyatta National Hospital, Nairobi, Kenya

PROTOCOL OUTLINE

**Title**	Feasibility of management of apnea of prematurity with caffeine citrate at a tertiary health care facility in Kenya. A quality improvement study.
**Rationale**	Neonatal mortality contributes about 50% of all deaths under age of five years out of which 36% are associated with prematurity. Majority of babies born before 34-weeks gestational age experience apnea with increased risk of death or adverse sequalae. Caffeine citrate is the recommended treatment for apnea of prematurity. A clinical feasibility pilot study of administration of caffeine citrate for at-risk neonates will be undertaken to inform a contextually appropriate clinical protocol as well as a broader implementation strategy.
**Population**	Healthcare providers and personnel, caregivers, and neonates at Kenyatta National Hospital involved in care of newborns with apnea of prematurity.
**Objectives**	**Phase I: Formative research**<br>To develop a context-appropriate prototype clinical care bundle, that includes caffeine citrate, for management of apnea of prematurity among neonates and an implementation strategy using quality evidence from key stakeholders' consensus opinion, and context-appropriate clinical data.<br><br>**Phase II: Pilot quality improvement implementation study**<br>To develop a scalable implementation strategy of a clinical care bundle, that includes caffeine citrate, to manage apnea of prematurity in neonates 34 weeks and below using a plan-do-study-act (PDSA) framework, a continuous quality improvement strategy.
**Endpoints**	1. Key metrics for adoption of and adherence to monitoring neonates and managing apnea of prematurity with caffeine citrate.<br>2. Identified barriers to and facilitators for effective implementation of the apnea of prematurity clinical care bundle.<br>3. Assessment of feasibility, usability, and acceptability of the apnea of prematurity clinical care bundle that includes monitoring of at-risk neonates and managing apnea of prematurity with caffeine citrate by healthcare administrators, healthcare providers, and caregivers.<br>4. Defined target quality improvement metrics associated with each plan-do-study-act cycle.<br>5. A context-appropriate apnea of prematurity clinical care bundle which is acceptable to stakeholders<br>6. Provide evidence to inform larger scale implementation science work in low- and middle-income countries.

Timeline	Total project anticipated to take 15 months to complete.

1 STRUCTURED ABSTRACT

Study background: At Kenyatta National Hospital, caffeine citrate is the recommended management for apnea of prematurity; however, it is not always implemented.
Broad objective: Undertake a clinical feasibility study on administration of caffeine citrate for neonates at risk of apnea of prematurity to inform a scalable, contextually appropriate clinical care bundle.
Study design and site: This will be a prospective mixed methods clinical feasibility study for quality improvement on caffeine use for treatment of apnea of prematutity to be conducted at the Kenyatta National Hospital newborn unit.
Participants and methods: Study participants will be health care providers, health care administrators and care givers/mothers of preterm newborn babies at Kenyatta National Hospital. In the first part of the study, care management of neonates with apnea of prematurity admitted at the newborn unit will be observed and their clinical data collected while on 'normal standard' treatment, that may or not include caffeine. Health care providers, care givers and health care administrators will also be interviewed to identify enablers and challenges to delivery of optimal care. The data will then be used to develop a context appropriate clinical care bundle that includes caffeine citrate in treatment of apnea of prematurity. This will be administered to all eligible babies uniformly in the second part of the study. A plan-do-study-act framework will be used to develop, optimize and pilot implementation of the bundle. The quality improvement strategy will examine adoptability, feasibility, usability, acceptability, adherence, and accessibility by target users.
Data management: Data related to study endpoints will be recorded or uploaded to and then extracted from electronic databases. All data will be maintained through a combination of a secure and access-restricted electronic data management system (REDCap) and physical files with restricted access. For qualitative data, NVIVO software will be used to organize, code, and analyze in an iterative process using thematic content analysis and triangulation. Quantitative data will be presented as frequencies and proportions for categorical data and as means and standard deviations or medians and interquartile ranges for continuous data. Normality of the data for the continuous data will be analyzed using the Shapiro Wilk test. Univariate analysis will be performed when comparing two or more groups to analyze any associations with the risk factors. Chi-square test or Fishers exact test will be performed on categorical data and Students t-test or Mann Whitney test for continuous variables when comparing 2 or more groups. Multivariate logistic regression will be utitized to determine independent associations.
Study Significance: The apnea of prematurity clinical care bundle developed from this study will be recommended for adoption at Kenyatta national hospital and, after further implementation research, scale up in other public health facilities with similar setting realities.

2 BACKGROUND AND RATIONALE

Neonatal mortality remains high in resource-constrained settings. Globally, 47% of all child deaths under the age of 5 years occurred in newborns in 2019, up from 40% in 1990. In Kenya, the estimated neonatal mortality rate is 22/1000 live births (Kenya National Bureau of Statistics, et al, 2015). Most deaths occur within the first few weeks of newborn life, mainly from preterm birth complications (especially in neonates born at less than 34 weeks' gestation), intrapartum-related complications, and infections, which can quickly progress to critical illness. A common clinical finding associated with increased vulnerability in newborns, especially in

preterm newborns, is apnea. Apnea or other types of ineffective breathing can lead to hypoxemia and bradycardia that may cause severe long-term disability, or if untreated, may result in death.

To increase respiratory drive and reduce apnea and its consequences, caffeine citrate is the recommended treatment of choice. With demonstrated lower toxicity, caffeine is superior to aminophylline in improving efficacy of supplemental oxygen and reducing incidence of complications (Zhang, et al., 2020). In addition to decreasing the risk of bronchopulmonary dysplasia, caffeine is neuroprotective and associated with improved cognitive outcomes at 2 years of age (Moschino, et al., 2020). Caffeine is also associated with improved pulmonary function up to 11 years of age.

Problem Statement

Despite being used widely in the United States and elsewhere both as treatment for and prevention of apnea of prematurity, caffeine is not routinely used in resource-constrained settings. Primary barriers to caffeine use include high drug prices (30mg/3 mL vial: $1.73 in Ghana to $73.63 in Kenya) and a lack of drug availability for purchase (Ekhaguere, et al., 2020). Furthermore, identifying neonates at risk for apnea and those requiring prophylaxis or treatment with caffeine is challenging in resource-constrained settings where gestational age is not always known and continuous monitoring of sick neonates is not routinely available.

The recent World Health Organization (WHO) standards for improving quality of care for small and sick newborns in health facilities has prioritized the assessment and management of apnea (WHO, 2020).

Currently at KNH, caffeine is recommended for prevention of apnea of prematurity for all preterm neonates less than 34 weeks – administered until attainment of 34 weeks corrected gestational age gestation (Kenyan Ministry of Health, 2018). When caffeine is unavailable, aminophylline is used as an alternative as it is less expensive and more readily available. Consequently, aminophylline is more widely used for prevention and treatment of apnea of prematurity in public health facilities in Kenya.

Assessment and management of apnea in small and sick newborns requires adequate and appropriate monitoring, however, this is not routinely done in many resource limited settings, largely due to unavailability of pulse oximeter devices. For example, the current practice at KNH is to perform intermittent spot-checks with pulse oximetry when devices are available. This approach may fail to identify many episodes of apnea of prematurity except in the neonatal intensive care unit where continuous monitoring is more reliably performed. Even for those on recommended monitoring, there is no standardization of clinical care protocol to be uniformly followed at KNH and other health care facilities in the country. There is thus need to develop and implement evidence-based and context-appropriate clinical guidelines for standardization and improvement of care to these babies. Challenges with implementation of such clinical guidelines include questions around optimal timing and dosage of caffeine therapy, standardization of alarm limits, and discharge monitoring protocols, among others (Eichenwald, 2020; Moschino, et al., 2020; Conlon, et al., 2021).

As part of the Evaluation of Technologies for Neonates in Africa (ETNA) platform in Nairobi, Kenya, we propose to include continuous or intermittent physiological monitoring during the period of piloting the implementation strategy for the clinical bundle for prevention and treatment of apnea that includes caffeine citrate. This will improve chances of detecting apnea of prematurity among the study participants.

2.1 Study justification

This study will provide important evidence to help inform larger-scale implementation science work that could make a strong case for improved availability and efficient use of caffeine in low- and middle-income country settings. Health systems are complex, thus while making caffeine available to improve prevention and treatment of apnea of prematurity, this in practice may not be the case. It is therefore crucial to have an understanding of how the health system, including the hospital setting, works by observing it before any intervention is carried out and identify barriers to and facilitators for appropriate use of caffeine. This information will help develop context sensitive apnea clinical bundle and an implementation strategy that will mitigate potential barrriers and build on the facilitators for the adoption of the bundle.

KNH is a training facility for paediatric residents and neonatal nurses who have experience in working in different regions in the country. Thus, engaging the KNH staff in a mulitidisciplinary continuous quality improvement approach to identify barriers and device pragmatic solutions by bringing onboard this wide representation with views and experiences from wide range of health facilities' settings will facilitate scalability of the bundle.

3 STUDY HYPOTHESIS, OBJECTIVES, AND ENDPOINTS

3.1 Hypothesis

We hypothesize that an evidence-based, accessible, simple-to-use and well-accepted clinical care bundle incorporating caffeine citrate in the prevention and treatment of apnea of prematurity will lead to better compliance with best practices in the management of neonates with apnea of prematurity at a tertiary health care facility in Kenya.

3.2 Objectives

Phase I: Formative research

To develop a context-appropriate prototype clinical care bundle, that includes caffeine citrate, for management of apnea of prematurity among neonates and an implementation strategy using quality evidence from key stakeholders' consensus opinion, and context-appropriate clinical data.

Phase II: Pilot implementation study

To develop a scalable implementation strategy of a clinical care bundle, that includes caffeine citrate, to manage apnea of prematurity in neonates 34 weeks and below using a plan-do-study-act (PDSA) framework, a continuous quality improvement strategy.

Endpoints

This project will support the adoption of best practices in the prevention and treatment of apnea of prematurity. Key practices will include how to monitor neonates and manage apnea of prematurity with caffeine citrate using a context-appropriate and well accepted clinical care bundle. Endpoints of this feasibility study are:

1. Key metrics for adoption of and adherence to monitoring neonates and managing apnea of prematurity with caffeine citrate.
2. Identified barriers to and facilitators for effective implementation of the apnea of prematurity clinical care bundle.
3. Assessment of feasibility, usability, and acceptability of the apnea of prematurity clinical care bundle that includes monitoring of at-risk neonates and managing apnea of prematurity with caffeine citrate by healthcare administrators, healthcare providers, and caregivers.

4. Defined target quality improvement metrics associated with each plan –do-study-act cycle.
5. A context-appropriate apnea of prematurity clinical care bundle which is accepted by stakeholders
6. Provide evidence to inform larger scale implementation science work in low-and middle-income countries.

4 METHODS

4.1 Study site

This project will be implemented at the Kenyatta National Hospital (KNH) newborn unit by a team of KNH and Aga Khan University (AKU) investigators supported by co-investigators from the University of Washington and University of British Columbia.

KNH is Kenya's premier national medical teaching and referral hospital with a busy newborn unit and experienced neonatologists. KNH is a public tertiary hospital and receives referrals from both within and outside Kenya in the Eastern and Central African region. The KNH newborn unit admits on average 150 neonates per month. About 13% of neonates admitted to the newborn unit are very low birth weight (1000-1499 grams), with 85% of these neonates being admitted on their day of birth. The newborn unit has a 60-bed capacity, though average bed occupancy is about 150%. Staffing in the newborn unit includes 5 neonatologists, 7 neonatology fellows, 2 pediatricians, and 12-15 pediatric residents. The unit has a compliment of about 80 nurses working in mixed shifts with an average of 13 nurses on duty at any time. Primary reasons for admissions to the KNH newborn unit include intrapartum-related complications, respiratory distress syndrome, neonatal sepsis, low birthweight, and neonatal jaundice.

The KNH newborn unit joined the Clinical Information Network (CIN) in 2019. The mission of CIN is to assist hospitals to generate quality patient data and analyze and improve data utilization (Tuti, et al., 2016) for improvement of services. The KNH newborn unit implemented the Newborn Essential Solution and Technology (NEST) Program in June 2021. This program provides the essential technologies (including CPAP, oxygen flowrate splitters, pulse oximeters, LED phototherapy lights and light meters, newborn glucometers, and radiant warmers with temperature sensors) and builds capacity for hospital staff to provide secondary level newborn unit care. Clinical practice guidelines in use include Ministry of Health guidelines for tertiary level care and for secondary level newborn care, such as comprehensive newborn care protocols that integrate clinical care pathways with guidance on the use of newborn care technologies.

4.2 Study design

This protocol details a prospective mixed methods clinical feasibility study on the use of caffeine citrate for the management of apnea of prematurity in a single facility, KNH, to inform development of a contextually appropriate apnea of prematurity clinical care bundle as well as a broader implementation strategy for quality-of-care improvement. The project will include a formative data collection phase followed by a quality improvement implementation phase that will incorporate evidence and lessons identification from earlier phase in the care bundle for neonates at-risk of apnea of prematurity. The following approaches will be used in phase I to obtain evidence that will inform the implementation phase of the study:

1. Systematic review of published literature for available best evidence to inform care bundle.
2. Qualitative research, including onsite observations, in-depth interviews (IDI), and/or focus group discussions (FGD) with key stakeholders (e.g., HCAs, HCPs, and caregivers).
3. Quantitative research will include collection of neonatal patient-level data.

The formative research phase will take about four months.

4.3 Study population

This pragmatic study design will engage key stakeholders, including KNH newborn unit HCPs (e.g., qualified nurses, physicians, neonatology fellows, and residents) and personnel (e.g., unit administrators, support staff, etc.), caregivers, and neonatal patients admitted to the newborn unit during the study period. Key stakeholders external to the newborn unit (e.g., HCAs, drug suppliers, hospital supplies department, pharmacists, etc.) may also be engaged. All engaged stakeholders will be aged 18 years or older and will have provided written informed consent. The HCPs will need to have been involved in newborn care for over three months preceding this period of data collection.

Neonates at-risk for apnea of prematurity admitted to the KNH newborn unit will be eligible for inclusion. Neonates will be considered at-risk if (i) born less than 34 weeks' gestational age or (ii) birthweight less than 1500 grams where the gestational age is unknown, provided estimated gestational age is less than 34 weeks as determined using the New Ballard Scoring for gestational assessment as per current practice at KNH. Neonates who receive aminophylline or caffeine citrate treatment as per current KNH practice will be enrolled and followed for 7 days after discontinuation of aminophylline or caffeine citrate treatment by the primary physician or up to 35 weeks corrected gestational age, whichever occurs later. Similarly, at-risk neonates who have an indication for management with aminophylline or caffeine citrate treatment as per current guidelines but for whom aminophylline or caffeine citrate treatment will not have been initiated, will also be followed for a minimum of 7 days after enrollment or longer if they develop apnea (until 48 hours free of any documented/reported apneic episode). Enrolled neonates with a gestational age greater than 34 weeks who receive aminophylline or caffeine citrate for prevention or treatment of apnea will also be followed for 7 days after the treatment is discontinued. Follow-up for at-risk newborns will be continue as described even when level of care is escalated (e.g., to CPAP or mechanical ventilation).

4.4 Sample size

For the quantitative patient data, we shall enroll consecutive patients who meet the inclusion criteria. Thus the sample size for enrollment in both phases of the study will be determined by the number of neonates admitted to the KNH newborn unit during the defined study periods and who also meet study eligibility criteria.

Data collection methods for the qualitative study will include onsite observations, IDI, and FGD. Four FGD will be conducted with HCPs and HCAs and four FGD will be conducted with caregivers with each FGD having 7-11 respondents. Overall a minimum of 12 and a maximum of 30 HCPs and HCAs and a minimum of 12 and a maximum of 18 caregivers will be recruited to participate in the study.

IDI will be conducted with the in-charges of the transdisciplinary units that directly or indirectly offer or influence the services delivered in the newborn unit. These may include the newborn unit in-charge (e.g., nursing officer, neonatologist), in-charge of pharmacy, hospital HCAs, hospital supplies department, and drug suppliers as will be consultatively defined prior to commencement of the interviews. Other key stakeholders will be identified by a snowball sampling approach.

Guided by a checklist, onsite observations will be carried out over a two-week period at the beginning of the formative phase of the study. Iterative analyses of the data will be performed with additional stakeholders identified by snowball sampling until data saturation is reached.

STUDY PROCEDURES

Refer to Appendix I for Schedule of Study Procedures and Evaluations.

4.5 PHASE I: FORMATIVE RESEARCH

This phase will commence with a systematic review of published literature on the management of apnea of prematurity with caffeine citrate. In addition, we will review the literature with special focus on eliciting the ethical issues that concern the subsequent implementation phase and sustainability of best practices after the study. We will then undertake formative observational research to assess barriers to and facilitators for caffeine citrate uptake within the KNH newborn unit and in the wider healthcare system. Current use of aminophylline and caffeine for apnea of prematurity management within routine clinical practice will be studied to provide information for eventual incorporation in a context-appropriate apnea of prematurity clinical care bundle. The aims of this formative research will be to identify barriers to and facilitators for the appropriate use of caffeine citrate in preventing and treating apnea and to provide evidence-based guidance for an apnea of prematurity clinical care bundle that includes identification of at-risk neonates, the rational use of caffeine citrate, monitoring of at-risk neonates, and clinical considerations for initiation and discontinuation of caffeine citrate.

4.5.1 Neonate screening

All newborns at risk of apnea of prematurity admitted to the newborn unit during the period of the formative study will be considered for inclusion. A brief introduction to the study will be provided to the caregiver(s) and informed written consent for data collection and use will be obtained (refer to Appendix II for Informed Consent). Screening will be conducted by KNH nurses as per recommended standard practice in the KNH newborn unit. Anonymized information and data will be collected. For those neonates whose parents decline to consent to have their data included, study staff will assure the caregiver(s) that their neonates will continue to receive KNH standard care and will not be treated differently from all other neonates undergoing care in the unit. There will be no preferential treatment of study participants. All screening procedures will be documented in the appropriate study logs and data collection forms (DCF). Clinical assessments and findings will also be documented. No identifying information will be retained for any neonate whose caregivers decline study participation. All neonates, whether enrolled in the study for data collection or not, will continue to receive KNH standard care.

4.5.2 Informed consent process

Informed consent will be obtained from each neonate's caregiver after ensuring that the caregiver is fully informed and understands what information will be collected regarding their neonate(s) and themselves and how the data will be stored and used. Trained study staff will administer a comprehension checklist to potential participants' caregivers prior to obtaining written informed consent. Data of neonates whose caregivers decline to participate in the qualitative study interviews will be included in the study analysis unless the care giver declines

consent for that too. Copies of signed informed consent will be kept in the participant's study binder and patient's hospital file while the third copy will be issued to the caregiver.

More derails on the consenting process are available in Appendix I.

4.5.3 Enrollment of neonates

After screening is complete, trained study staff will perform the study enrollment procedures for those eligible neonates whose caregiver has provided written informed consent and meet Inclusion Criteria as follows:

- Neonates admitted to the KNH newborn unit during the study period who are born less than 34 weeks gestational age or with a birthweight less than 1500 grams where the gestational age is unknown and who are estimated to have a gestational age less than 34 weeks by New Ballard Scoring for gestational assessment as per current practice at KNH;
- Neonates on either aminophylline or caffeine citrate based on existing clinical criteria or clinical events;

Exclusion Criteria:

- Unwillingness or inability of neonate's caregiver to provide informed consent for data collection and use.

The following procedures will be performed at enrollment:

- Assign participant identification (PID) study number.
- Collect gestational age/corrected neonatal age, sex, weight, other patient, birth, maternal, and family characteristics, any comorbidities and medical history, and any additional socio-demographic information not already collected during screening.
- Collect information regarding duration of pregnancy, mode of delivery, and Apgar score results.
- Collect information regarding medications, treatments, and monitoring for the neonates.
- Obtain baseline vital signs, number and frequency of apneic episodes (if any).
- Collect information on dosing (dose and frequency), timing, and duration of aminophylline or caffeine citrate treatment if the neonate is on this treatment.
- Collect information on timing and duration of monitoring of vital signs, apneic episodes, clinical conditions and co-morbidities (if any).

All study enrollment procedures will be documented in the appropriate DCF. Clinical assessments and findings will also be extracted from the enrolled neonate's medical record.

4.5.4 Neonate data collection

Enrolled neonates will be followed for 7 days after the discontinuation of aminophylline or caffeine citrate treatment as determined by the primary physician or up to 35 weeks corrected gestational age, whichever occurs later. If aminophylline or caffeine citrate treatment is indicated per current guidelines but not initiated, or if aminophylline or caffeine citrate is given to neonates born over 34 weeks' gestational age, observation of those at-risk neonates will be undertaken for 7 days after discontinuation of treatment. Data will continue to be collected from neonates who undergo escalation of care to CPAP or mechanical ventilation.

Data on an enrolled neonate's progress and follow-up care will be extracted from the neonate's clinical records, monitoring device(s), and treatment charts. This will be complemented by other

sources of relevant patient data such as existing databases or other ongoing studies at the site, in accordance with approved study protocols. Study staff will also observe and record their findings. The source documents will be reviewed and weight, vital signs, oxygen utilization, treatments, new diagnoses, clinically significant events, patient outcomes at the end of hospital stay and significant observations will be entered in the DCF. Data will be collected daily during the follow-up study period.

Observations will continue while the enrolled neonate is receiving clinical care until the study follow-up period is over. During observations, the following will be done:

- Recording of daily weight.
- Updating of medical history, including new diagnoses, clinically significant events, clinical procedures, interventions (e.g., intravenous line insertion, naso/oral gastric tube placement, etc.), medications, therapies, additional bloodwork or laboratory tests and any other relevant clinical characteristics.
- Extracting data from neonatal patient charts to update information regarding number, frequency, causes and interventions related to apneic episodes.
- Collecting information about dosing, timing, duration, and changes to aminophylline or caffeine citrate treatment.
- Collecting information about timing and duration of monitoring of respiratory rate, heart rate, oxygen saturation, duration of apneic episodes, and comorbidities.
- Collecting information on utilization of oxygen (and fraction of inspired oxygen when blending is provided) and/or CPAP or other ventilatory support requirements at point of initiation and discontinuation of caffeine.
- Assessing and reporting of adverse events to ongoing treatments.
- Any other observations considered relevant to the study.

4.5.5 Stakeholder qualitative data collection

A critical aspect of the formative research phase will be structured engagement with key stakeholders, both internal and external to the newborn unit, to identify critical barriers to and facilitators for the availability and use of caffeine citrate within the unit.

The following categories of stakeholders will be included:

- KNH newborn unit stakeholders, including HCPs (e.g., trained nurses, physicians, neonatology fellows, and residents), HCAs, support staff involved in the care of neonates, and caregivers who have a neonate enrolled in the neonate cohort.
- KNH newborn unit external stakeholders, including hospital HCAs, hospital supplies officers, drug suppliers, and pharmacists involved in the procurement, preparation and supply of aminophylline or caffeine citrate.

Qualitative data collection will be conducted by a qualitative research team led by a social scientist with experience in conducting and analyzing qualitative research. This study will employ onsite observations, IDI, and FGD. IDI will be conducted with the in-charges of the transdisciplinary units that directly or indirectly offer or influence the services delivered in the newborn unit. These may include the newborn unit in-charge (e.g., nursing officer, neonatologist, neonatal fellow), in-charge of pharmacy, hospital HCAs, hospital supplies department, and drug suppliers. Other key stakeholders will be identified by a snowball sampling approach, and data will be analyzed through thematic content analysis and triangulation of data from different sources. Data collection will continue until saturation is

reached. IDI and/or FGD will be conducted with HCPs working in the KNH newborn unit and with caregivers of neonates enrolled in this study. Each FGD will consist of a heterogeneous group of the respondents. Caregiver FGD will comprise of fathers and mothers of varying ages and educational status. Similarly, HCP groups will include different cadres of HCP of varying age and gender. We envisage that heterogenous groups will yield rich discussions. In both IDI and FGD, the focus will be on systems rather than on behavior of individuals and the approach will be non-judgemental. This is in recognition that behavior that is not consistent with the uptake of best practices could be ‘maladaption’ to an inefficient health system (Irimu, et al., 2014). Using a checklist, trained research assistants will conduct observations of HCPs in the KNH newborn unit over a two-week period to provide contextualized insights on actual care practices within the newborn unit.

Informed consent to participate in onsite observations, IDI, and/or FGD and to be audio-recorded will be obtained. IDI and FGD will be audio-recorded and transcribed verbatim while noting the body language. IDI and FGD will be conducted by an interviewer (IDI) or moderator (FGD) assisted by a notetaker. The notetaker will be in charge of notetaking, audio-recording and recording of any body language relevant to the discussions and themes of the study. IDI and FGD guides will be developed and used to conduct the sessions and will contain broad questions whose order can be adjusted based on the informants and contexts. FGD will take between 60-90 minutes while IDI are estimated to take between 45-60 minutes. Confidentiality will be maintained during the discussions. Participants will also sign a non-disclosure clause within the informed consent form (ICF) that prohibits discussions of the proceedings of the FGD outside of the group. Data from the discussion in the form of audio-recordings, transcripts, and field notes will be treated confidentially and stored in laptops with a password and in secured lockers at AKU. They will not be accessible outside the study team.

4.5.6 Transition from formative research phase to quality improvement implementation phase

The primary output of the formative research phase will be a prototype apnea of prematurity clinical care bundle and an implementation strategy for use in the subsequent pilot quality improvement implementation phase. This will involve ‘fact finding’ and planning for change discussions. Focus will be on overcoming the barriers and encouraging the facilitators identified during the formative research phase. Implementation study questions and quality improvement interventions will be identified and developed during the formative research phase. This will help define apnea management criteria, caffeine citrate formulation, dosage, route of administration, monitoring requirements, and criteria for weaning and discontinuation. Review of policies, standard operating procedures, guidelines, publications, and practices around the prevention and treatment of apnea of prematurity will also be undertaken. Additionally, we will identify early adopters and potential “champions” to enlist in the pilot quality improvement implementation phase. An understanding of the determinants of implementation will be critical to implementation strategy development.

A training program will be developed for implementing and testing of the apnea of premaurity clinical care bundle in the KNH newborn unit. Relevant trainings will be given to all those directly involved in healthcare service delivery to premature and low birthweight neonates at KNH. This will include KNH newborn unit HCPs, pharmacists, health records and information officers (data entry personnel), and other personnel identified to be of relevance to the process by the hospital staff.

4.6 PHASE II. QUALITY IMPROVEMENT IMPLEMENTATION

We will use a quality improvement approach that utilizes a theoretical framework based on the Berwick Rules (Berwick, et al., 2003). This will start with piloting the prototype apnea of prematurity clinical care bundle developed during the formative research phase using the pre-defined implementation strategy. Over a 4-month period, we will perform multiple cycles of quality improvement following a PDSA framework, ensuring that recommended changes are tested, adapted to context, and accepted by users. The following parameters will be considered in the assessment of feasibility of the apnea of prematurity clinical care bundle and implementation strategy (Pearson, et al., 2020):

1. Adoption: the proportion of eligible neonates who receive the apnea of prematurity clinical care bundle.
2. Fidelity: adherence to the apnea of prematurity clinical care bundle based on the number and severity of deviations.
3. Acceptability: stakeholder satisfaction with the apnea of prematurity clinical care bundle.
4. Adaptability: ability to adapt the apnea of prematurity clinical care bundle to local needs.
5. Appropriateness: alignment of the apnea of prematurity clinical care bundle with organizational and individual values, mission, and priorities.
6. Complexity: degree of difficulty or effort in administering the apnea of prematurity clinical care bundle.
7. Culture: political, economic, or institutional norms, values, or assumptions influencing adoption of the apnea of prematurity clinical care bundle.

Our trained team of study nurses under the supervision of the study investigators will work closely with KNH hospital staff and leadership using workflow management tools with pre-set key performance indicators agreed *a priori* with the hospital staff. The key focus will be to have a continuous quality improvement approach that will identify and then overcome barriers to and/or encourage facilitators for adoption that can be used to inform a larger-scale multi-site intervention. A PDSA framework will be used to promote the adoption of the prototype apnea of prematurity clinical care bundle and implementation strategy that will be continually refined to be contextually feasible, usable, and acceptable in the study setting.

We recognize that simply having an apnea of prematurity clinical care bundle and implementation strategy does not lead to change; rather, attention and effort will be required to have a system that supports change by building on the facilitators for and mitigating potential barriers to adoption of the bundle. We plan to primarily use the Capability, Opportunity, and Motivation model of behavior change (COM-B; Michie, et al., 2014).

Apneic episodes will be identified through monitoring the neonate's clinical condition and vital signs continuously upon initiation of caffeine citrate for at least 48 hours or 48 hours after cessation of any identified apneic events unless evidence from the formative research phase suggests otherwise. To avoid confusion with different formulations, only one caffeine citrate brand will be in use at the newborn unit during the study period. This will be provided to all neonates receiving apnea of prematurity treatment during the quality improvement implementation phase. Any support identified in phase I will be provided through KNH leadership and the newborn unit to facilitate uptake (and minimize barriers) of the apnea of prematurity clinical care bundle and implementation.

4.6.1 Neonate screening, informed consent process, neonate enrollment, neonate data collection

Neonate screening, informed consent process, and neonate enrollment for the formative research phase is described above. Since caffeine citrate is the standard of care treatment for apnea of prematurity at KNH, informed consent will not be obtained before treatment but will be obtained for data collection and data use only. All other study procedures in the quality improvement implementation phase constitute recommended standard of care for neonates with apnea of prematurity summarized in a contextualized care pathway designed with the KNH staff. Caffeine citrate treatment will be prescribed by the attending physician.

Upon enrollment to the pilot quality improvement implementation phase, all enrolled neonates will receive continuous monitoring of heart rate, oxygen saturation, and apneic episodes with a monitoring device that will be connected to the neonate by a trained nurse following standard operating procedures established during the formative research phase of the study. This monitoring device will be a device approved for use in neonates with commercial availability in Kenya. The device will have the ability to digitally record the heart rate and oxygen saturation, and to provide user-adjustable apnea alerts for documentation of apnea. Detection of apnea will be characterized by the cessation of breathing for 20 seconds, associated with desaturation and bradycardia. Additionally, the admitting clinicians will be encouraged to perform and document New Ballard Scoring for gestational assessment and Silverman-Anderson respiratory severity scoring. These data will be supplemented with the clinical data extracted from the enrolled neonate's charts as described in the formative research phase. All enrolled neonates will have the apnea of prematurity clinical care bundle applied in a standardized method as per standard operating procedures. During the pilot quality improvement implementation phase, clinical patient data will be collected from study monitoring devices, neonatal DCF, patient chart reviews, and relevant checklists from enrolled neonates. Data on enrolled neonates will also be collected from other patient databases and ongoing studies, in accordance with all approved study protocols.

The quality improvement process will have several components:

(i) During the quality improvement implementation phase, different approaches will be used to strengthen the quality of routinely collected data. This will be done by providing regular audit reports focusing on quality of documentation and performance of healthcare workers regarding uptake of the apnea of prematurity clinical care bundle and performance of key processes and outcomes. Routine data collection, analysis and feedback will apply to all at-risk neonates admitted to the KNH newborn unit. Patient-level data will be captured, stored, and tracked through a Research Electronic Data Capture (REDCap) system.

(ii) Regular feedback on routine quality of care for all at-risk neonates, including caffeine citrate utilization will be provided. These outcomes will be reported using descriptive statistics of neonatal demographics and clinical interventions or events (such as frequency and duration of caffeine citrate use and other drugs, number and duration of apneic, cyanotic, or cardiac arrest/near arrest episodes, etc.). This will help in establishing proportions of neonates whose care is consistent with the accepted best practices for prevention and treatment of apnea of prematurity and the trend of uptake of the apnea of prematurity clinical care bundle.

(iii) Additionally, study-specific clinical patient data and data recorded from the study monitoring devices will be analyzed to provide an assessment of performance of key processes and outcomes. Surrogate indicators of quality of care will include incidence of apnea, level and duration of hypoxemia and hyperoxia, escalation of care, number and type of critical events, need for co-interventions, availability and utilization of supplies, etc.

(iv) Systematic case reviews will be conducted to identify inconsistencies in indications of caffeine citrate treatment, neonate assessment, neoante monitoring and management plans. A multidisciplinary clinical audit committee will be established with guidelines on how to conduct case reviews and audit procedures adapted from the WHO hospital-based guidelines for case reviews (WHO, 2018). Clinical audit meetings will be multidisciplinary and all those who deliver or influence adoption of the apnea of prematurity clinical care pathway at KNH will be invited, including newborn unit stakeholders (e.g., HCPs, HCAs, support staff involved in the care of neonates) and those external to the newborn unit (e.g., hospital HCAs, hospital supplies department, drug suppliers, and pharmacists). Principles of root cause analysis will be applied to help identify problems and potential solutions. KNH staff will then implement the solutions identified during the audit meetings. The practice to change, the level at which change should occur, the person(s) responsible, the resources required, and the timelines will be agreed upon during the audit meetings. Implementation of the audit processes such as frequency of audit meetings and selection criteria of neonates to be audited will be determined in consultation with the hospital team during the formative research phase. The process of conducting the audit meeting will be agreed with the hospital staff during the formative research phase, including duration, venue, frequency, and methods of inviting the participants as per the WHO hospital-based guidelines for case reviews (WHO, 2018). Confidentiality will be maintained during the discussions. Participants will also sign a non-disclosure clause as per the WHO guidance on conducting audits that prohibits discussions of the proceedings of the clinical meeting outside of the group. Data from all the discussions and interviews in the form of audio-recordings and transcripts will be treated confidentially as per hospital policy. Data will be analyzed qualitatively to assess the feasibility, acceptability and usability of the apnea of prematurity clinical care bundle and the results used to refine the implementation strategy developed in the formative phase.

(v) PDSA processes will be repeated in cycles of quality improvement to refine the apnea of prematurity clinical care bundle and the implementation strategy to help facilitate adoption. At each cycle of quality improvement, we will work with stakeholders to identify elements for improvement. The process will be repeated to maintain continuous system improvement characterized by satisfactory uptake of best practices in apnea of prematurity management. Our qualitative team will join in the audit meetings and help in problem analysis and solution identification where appropriate. Satisfaction regarding the apnea of prematurity clinical care bundle will be explored during the multidisciplinary clinical audit meeting.

5 TIMELINE

This project was anticipated to be implemented over a 15-month time period from October 2021 to December 2022 with the formative phase commencing in January 2022, but actual dates would depend on when ethical approvals will be received. Additional time may be required in event of disruption of implementation. There will be a 4-month project start-up phase. The

formative research phase will run for 4 months and then the pilot quality improvement implementation phase will run for another 4 months. As data are collected, data analysis and synthesis will be ongoing throughout the project. Stakeholders' analyses and identification of barriers and facilitators will be concurrently studied during the formative research phase to inform design of the apnea of prematurity clinical care bundle to be tested during the quality improvement implementation phase through PDSA cycles. The last 3 months of the project will be used to complete data analysis, synthesize, and disseminate the findings.

	**Year 1**				**Y2**
	Q1	Q2	Q3	Q4	Q1
**1. Project start-up**	■	■			
Develop protocols and materials	■				
Hire and train staff	■				
Establish sub-agreements and sub-awards	■	■			
Assess Kenyan caffeine citrate supply chain	■	■			
Purchase supplies (pulse oximeters, caffeine citrate for implementation phase)		■			
Obtain clearances and ethical approvals		■			
Develop tools, operations and communications systems		■			
**2. Formative research phase**		■	■		
Engage with facility-based (clinicians, pharmacists, administration, etc.) stakeholders		■	■		
Identify current practices, barriers to, and facilitators for caffeine citrate in facility		■	■		
Identify early adopters/champions		■	■		
Conduct facility-based baseline data collection, enroll and monitor neonates		■	■		
Analyze formative research data		■	■		
Develop apnea of prematurity clinical care bundle with a detailed implementation plan		■	■		
**3. Quality improvement implementation phase (utilizing PDSA approach)**			■	■	
Quality improvement cycles			■	■	
Conduct feasibility, usability, acceptability evaluation			■	■	
Analyze quality improvement data			■	■	
**4. Knowledge translation**					■
Synthesize findings, share data, and build consensus with stakeholders					■
Develop apnea of prematurity clinical care bundle					■
Disseminate findings and consider further implementation research recommendations					■

6 STUDY PROCEDURES

Refer to Appendix I for Schedule of Study Procedures and Evaluations.

6.1 Qualitative evaluations

Formal qualitative analysis will evaluate feasibility, usability, and acceptability of the apnea of prematurity clinical care bundle. Qualitative data collection will be conducted by a qualitative research team and will include onsite observations, IDI, and FGD during the formative research phase and the last quality improvement cycle of the implementation phase.

6.2 Withdrawal and early termination

Neonates and their caregivers may voluntarily withdraw from the study for any reason at any time. The study investigators may also withdraw neonates from the study to protect their safety if, in the investigators' opinion, continuing participation would jeopardize the neonate's health. HCPs, HCAs, and caregivers may voluntarily withdraw from the qualitative portion of the study for any reason at any time. Any participant withdrawal or early termination will be documented in the appropriate study forms.

6.3 Study termination

Neonate study participapants will be discharged from the study after minimum of 7 days from discontinuation of caffeine unless PDSA findings otherwise dictate. Study staff will liaise with the hospital staff to be notified of when a participant will be discharged. At the conclusion of participation, the following procedures will be conducted:

- Record weight.
- Update clinically significant events.
- Update medical history, including new diagnoses or any comorbidities, clinical procedures, interventions (e.g., kangaroo mother care, repositioning, line insertion, nasogastric tube placement, umbilical wound care, UV therapy, stimulation, etc.), medications, therapies, additional bloodwork, or laboratory tests.
- Update information regarding number and frequency of apneic episodes.
- Collect information about discontinuation of aminophylline or caffeine citrate treatment.
- Collect information about discontinuation of monitoring.
- Collect information about discontinuation of oxygen and/or CPAP or other ventilatory support requirements.
- Record duration of hospitalization.
- Assess for safety issues and report safety events.
- Document contact in neonate's study records.

7 STUDY STAFF TRAINING REQUIREMENTS

All study staff will have valid certification in the Protection of Human Subjects and Good Clinical Practice (GCP) prior to any interactions with study participants. Also prior to study initiation, all study staff will receive study-specific training on any study tasks or procedures necessary to carry out their defined study role(s), including the study protocol, standard operating procedures, data collection tools, informed consent process and reporting, etc. Trainings will be conducted by a study PI or their designee, as appropriate for the training material.

Patient data will be captured, stored, and tracked in the REDCap data collection tool by study staff, whose training will be informed by the standard operating procedures for this study. The study staff will be trained on patient assessment of the parameters in the data collection tool. All hospital staff involved in study recruitment procedures will be trained in relevant study-specific procedures and certified in GCP. Each recruitment and referral interaction will be documented for study records.

8 DATA COLLECTION, MANAGEMENT, AND ANALYSIS

Data will be collected throughout Phases I and II while neonates are enrolled. Patient-level data will be collected by study staff during workdays and recorded from source documents. All clinical study data will be maintained through a combination of a secure and access-restricted electronic data management system (REDCap) and physical files with restricted access. Data related to study endpoints will be recorded or uploaded to and then extracted from electronic databases for statistical analysis. All documentation (paper-based or electronic) that has both personal identifiers and the PID will have highly restricted access and will be stored in a secure manner separately from other study data.

8.1 Data collection forms

Study data such as those data elements listed in Appendix III will be collected by study staff using designated source documents. Study data will be entered directly into the DCF as promptly as is feasible. Study staff will maintain source documents for each study participant at the study site. Source documentation will be available for review to ensure that the collected data are consistent with the DCF. Source documents, and other supporting documents (both electronic and paper-based) will be kept in a secure location and remain separate from PID information (name, address, etc.) to ensure confidentiality. GCP will be followed to ensure accurate, reliable, and consistent data collection.

8.2 Source documents

Source documents include but are not limited to:

- Signed ICFs.
- Documentation of the comprehension checklist.
- Documentation that includes dates and times of clinical observations.
- Clinical chart and notes.

Study investigators and staff will maintain, and store in a secure manner, all source documents throughout the study.

8.3 Data management and monitoring

Study data management will be conducted by study staff. Data management activities include DCF data entry and validation, data cleaning, database quality control, disaster recovery plans, preparation and submission of compliance reports to the funding agency, and preparation of the final study database. Initial data validation will occur within REDCap at the time of data entry (e.g., setting minimum and maximum values for weight and heart rate). Further data validation, cleaning, and quality control will be conducted on a daily basis. Missing data or data irregularities will be identified and sent to study staff for query and confirmation or correction after review of source documentation. An audit trail of all digital data entry, modification, and export will be maintained. Detailed notes will accompany any change or modification of patient data in the study database. A back-up copy of study-related clinical monitoring data will be saved on asecure, onsite access-limited external hard drive. De-identified data will be downloaded from the secure electronic data management system to support statistical analyses. All transfer of data for analysis will use password protection and end-to-end encryption. An audit trail will be maintained for any de-identified data leaving for external analysis.

8.4 Missing data

Throughout neonate enrollment and follow-up, study staff will be reviewing for data completeness. If a neonate's study-related data is not collected on the DCF, study staff will review the neonate's clinical chart to identify and add the missing data to the DCF. Should study-related data be found missing after a neonate has exited the study, study staff will identify the location of the neonate's clinical chart within the hospital and collect and add the missing data to the DCF. All reasonable efforts will be made to locate and add missing data. If an exited neonate's clinical chart cannot be located, study staff will report the situation to the study PI who will determine if the missing data is sufficient to remove the neonate from the study.

8.5 Data analysis

Summary statistics will be presented as frequencies and proportions for categorical data and as means and standard deviations or medians and interquartile ranges (where appropriate) for continuous data. Normality of the data for the continuous data will be analyzed using the Shapiro Wilk test. Univariate analysis will be performed when comparing two or more groups to analyze any associations with the risk factors. Chi-squared test or Fishers exact test will be performed on categorical data when comparing 2 or more groups whereas Students t-test or Mann Whitney test will be performed on continuous data when comparing 2 or more groups. Furthermore, multivariate logistic regression will be utlitized to determine independent associations after adjusting for confounding variables. Odds ratio and 95% confidence levels will be presented. A p value of less than 0.05 will be considered significant and all tests done will be a 2 tailed test.

8.6 Qualitative data collection

Qualitative data will be collected through IDI and FGD. Interviews will be audio-recorded and transcribed verbatim. Similar to the DCF, data from the questionnaires will be entered into a secure and access-restricted electronic database as promptly as is feasible. The paper copy of the questionnaire will be maintained as source document for each HCP, HCA, or caregiver enrolled in the qualitative portion of the study. No identifying information will be collected during IDI or FGD. Paper notes will be kept in a secure location and remain separate from PID information (name, address, etc.) to ensure confidentiality. GCP will be followed to ensure accurate, reliable, and consistent data collection

Qualitative data will be analyzed to assess feasibility, usability, and acceptability of monitoring neonates and managing apnea of prematurity with caffeine citrate among HCPs and hospital HCAs, and acceptability among caregivers of neonates. All IDI and/or FGD will be audio-recorded and transcribed to form the core qualitative data. Observations and fieldnotes will be used to augment and contextualize IDI and/or FGD data. The qualitative data will be in narrative format and the results will be descriptive. The questionnaires will be coded and analyzed using a codebook with identified themes, including feasibility of using monitoring technologies and caffeine citrate, barriers to and facilitators for use, and perceived value. Qualitative data analysis software will be used to organize, code, and analyze the qualitative data in an iterative process. The study team will start by identifying an initial set of codes and themes based on the categories from the IDI and/or FGD guides. During the coding process, attention will be paid to identifying emergent issues and themes that will be added to the codebook and included in the analysis. Responses from the IDI and/or FGD will be coded and discrepancies will be discussed and resolved for the final analysis and theme identification.

8.7 Data access

Study investigators and designees will maintain appropriate medical and research records for this study, in compliance with GCP, regulatory, sponsoring organization and institutional requirements for the protection of study participant confidentiality. De-identified data will be provided to the investigators to facilitate data cleaning and analysis. The study site will permit

authorized representatives of the sponsor and regulatory agencies to examine (and when required by applicable law, to copy) clinical records for the purposes of quality assurance reviews, audits and evaluation of the study safety and progress. User rights will be provided to PIs, co-investigators, and study staff at the level appropriate for each individual's job description. Study PIs and co-investigators not affiliated with AKU or KNH will complete a data transfer agreement prior to receiving study data.

8.8 Data storage

Study investigators and designees will maintain, and store securely, complete, accurate and current study records throughout the study. Study staff will retain all study records on site for at least five years after study closure. Study records will not be destroyed prior to receiving approval for record destruction from the sponsor. Applicable records include source documents, ICF, and notations of all contacts with study participants. At the completion of the study, de-identified data will be transferred to a public data repository to share with other internal and external researchers.

9 SAFETY MONITORING

The study PIs, co-investigators, and staff will be responsible for close safety monitoring of all study participants, and for alerting the protocol team if unexpected concerns arise. Standard operating procedures for identification and reporting of adverse events will be developed prior to initiation of the pilot quality improvement implementation phase. Study investigators will hold regular conference calls to monitor progress and ensure homogeneity and safety in protocol execution.

In addition, for this project, additional multiparameter continuous physiological monitoring (MCPM) devices will be purchased to complement the limited available equipment within the KNH newborn unit. The rationale for including these MCPM devices is that we anticipate that the apnea of prematurity consensus guidelines and care pathway will likely include the option to escalate monitoring to more than pulse oximetry for the highest risk neonates (although this will be informed during the formative research phase and expert opinion), and for the safety of the neonates, we do not want to be left without this capacity. The standard of care in high-resource settings is to monitor at-risk neonates with MCPM devices. Also critical, we will want to have the ability to continuously record the monitoring data for this subset of neonates to provide some justification for this more intensive monitoring. This equipment will be retained by KNH for routine patient use at end of the project.

10 ETHICAL CONSIDERATIONS

10.1 Principles for clinical research

This clinical study will be conducted in compliance with the protocol, GCP, and all applicable regulatory requirements and institutional review board (IRB) reviews. All study activities will follow the ethical principles of the Declaration of Helsinki. All study staff will be trained and certified in the protection of human subjects.

10.2 Institutional review boards and independent ethics committees

The IRB of record for this study is University of Nairobi (UoN)/KNH IRB. A copy of the protocol, proposed ICFs, other written participant information, and any proposed recruitment materials will be submitted to UoN/KNH IRB for written approval with copies to AKU institutional ethics review committee (ERC) for information and record. The protocol will be submitted to both

UoN/KNH and AKU ERCs for review and approval. The study investigators will submit and obtain approval from the IRB at their local institution before initiation of the study. AKU is responsible for ensuring that this protocol, ICFs, and any other study-related documents are approved by the two ERCs prior to implementation of the study. Any subsequent amendments to the protocol or other study-related documents will be approved by UoN/KNH ERC prior to implementation and report of such changes and approvals provided to AKU IERC and PPB in the stipulated progress reports. Any deviations from or violations of the protocol will be documented and reported to the appropriate IRB(s) by investigators as per terms of ethics approval.

Administrative approval to carry out the project will also be sought from the KNH leadership.

10.3 Informed consent documentation

In obtaining and documenting informed consent, the study investigators and their designees will comply with applicable local and domestic regulatory requirements and will adhere to GCP. English and Swahili versions of the ICF will be reviewed and approved by the appropriate ERCs/IRBs before use with study participants. The ICF will include the purpose of the study, a description of the procedures to be followed and the risks and benefits of participation. The informed consent process will give study participants all relevant information necessary to decide whether to participate, or to continue participation, in this study. Potential study participants will be permitted to ask questions and to exchange information freely with the study staff. If the study participant providing consent is illiterate, an independent witness (not member of the study team) will be present to verify to the study participant that all the information read aloud is contained in the ICF. In this instance, the study participants will thumbprint the ICF, which will be countersigned by the impartial witness.

Before a study participant are enrolled, it will be the study investigator's responsibility to ensure that informed consent is obtained after adequate explanation of the aims, methods, and potential risks and benefits of the study. The study staff obtaining consent will also sign and date the ICF. A signed and dated copy of the consent form will be given to the study participant and this will be documented in the participant's study record.

10.4 Study discontinuation

The study may be discontinued at any time by the protocol team, funding agency, regulatory authorities, or ERCs/IRBs.

10.5 RISKS, BENEFITS, AND CONSTRAINTS

10.5.1 Risks to participants

- Coercion

Caregivers may feel coerced or compelled to enroll in the study in order for their neonate to receive care within a research setting, which may be perceived as higher quality than the standard of care. During the informed consent process, study staff will emphasize that participation in the study is optional and strictly voluntary, and that the neonates will receive the same medical care whether enrolled in the study or not. Similarly, HCP may feel coerced or compelled to enroll in the study as part of their position or job. During the informed consent process, study staff will emphasize that participation in the study is optional and strictly voluntary, and that the HCP will neither be penalized nor rewarded professionally as a result of their participation in the study.

- Medical management

Participation in the study has the potential to compromise a neonate's inpatient care if study procedures are prioritized. The first phase of this study is fully observational hence no director encounter with the study subjects while the second phase is centered on improving quality of care of all children with apnea.

- Confidentiality

All study staff will ensure that study participant confidentiality is maintained at all times. Personal identifiers will not be included in any study reports. All study records will be kept confidentially undere lock and key in keeping with IRB regulations as well as national and local laws. Video recordings and photographs, if taken for any reason, will not include the participant's face and would only be done with written consent from the participants or their legal guardians. All study procedures will be conducted in such a manner as to protect participant privacy and confidentiality to the fullest extent possible.

10.5.2 Benefits to participants

There is no direct benefit to participants enrolled in this study. A consistent supply of cost-free caffeine citrate will be available during the study period to all neonates at risk of apnea of prematurity without discrimination. Patients requiring caffeine treatment will receive caffeine citrate as recommended by primary doctors even if their caregivers decline to have their data included in the study. In addition, extra U.S. FDA-approved continuous physiological monitoring devices will be available during the study and retained by KNH upon completion of the study.

10.5.3 Potential constraints

Anticipated challenges to the successful implementation of the study include:

- Delays in stakeholder engagement and feedback. Regular communication between the co-PIs, co-investigators, and key stakeholders along with close monitoring of study progress will help to anticipate, prepare for, and mitigate potential issues that would cause delays in adequate stakeholder engagement and feedback. Good rapport will be established with stakeholders and meeting schedules planned in advance to provide enough notice to participants. Consensus will be sought on most acceptable meeting times.
- Difficulty in recruitment of neonates. This study will be conducted at KNH, a high-volume tertiary hospital, to maximize enrollment. Sensitization and awareness creation about the study will be conducted in advance and maintained during the sudy period. essment prior to starting.

11 DISSEMINATION OF STUDY RESULTS

Results of this collaborative study will be presented and disseminated at international conference(s) and/or through open-access, published manuscript(s) with detailed descriptions of the background, methods, results, discussion and conclusions. The specific format and details of any potential conference abstract or manuscript will be in accordance with the requirements of the conference and/or peer-reviewed journal.

Datasets including study indicators (which may include summary reports of clinical characteristics and outcomes, pulse oximetry recordings, summary reports of desaturation/ bradycardia events, summary reports of caffeine utilization, etc.) and deidentified qualitative datasets may be made publicly available after the planned primary and secondary data analyses are completed but in compliance with stipulated national data protection laws.
Data transfer agreements will be signed between parties in accordance with host institutions' policy guidelines before sharing anonymized data with third parties.

12 REFERENCES

Berwick, D. M. (2003). Disseminating innovations in health care. *JAMA: The Journal of the American Medical Association*, *289*(15), 1969–1975. https://doi.org/10.1001/jama.289.15.1969

Ekhaguere, O. A., Ayede, A. I., & Ezeaka, C. V. (2020). Is caffeine available and affordable in low and middle-income countries? A survey in sub-Saharan Africa. *Seminars in Fetal & Neonatal Medicine*, *25*(6), 101182. https://doi.org/10.1016/j.siny.2020.101182

Eichenwald, E.C. (2020) National and international guidelines for neonatal caffeine use: Are they evidenced-based? Seminars in Fetal and Neonatal Medicine Dec;25(6):101177. doi: 10.1016/j.siny.2020.101177

Irimu, G. W., Greene, A., Gathara, D., Kihara, H., Maina, C., Mbori-Ngacha, D., Zurovac, D., Santau, M., Todd, J., & English, M. (2014). Explaining the uptake of paediatric guidelines in a Kenyan tertiary hospital--mixed methods research. *BMC Health Services Research*, *14*, 119. https://doi.org/10.1186/1472-6963-14-119

Kenyan Ministry of Health. (2018). *National Newborn Guidelines for Hospitals.* https://www.coursehero.com/file/57699613/NEWBORN-GUIDELINES-FINAL-PRINTED-N-ONLINEpdf/

Kenya National Bureau of Statistics, Ministry of Health/Kenya, National AIDS Control Council/Kenya, Kenya Medical Research Institute, and National Council for Population and Development/Kenya. (2015). Kenya Demographic and Health Survey 2014. http://dhsprogram.com/pubs/pdf/FR308/FR308.pdf

Michie, S., Atkins, L., West, R., & Others. (2014). The behaviour change wheel. *A Guide to Designing Interventions. 1st Ed. Great Britain: Silverback Publishing*, 1003–1010. https://www.researchgate.net/profile/Susan_Michie/publication/311857816_Changing_Behaviour_to_Improve_Clinical_Practice_and_Policy/links/588249b9aca272b7b4425460/Changing-Behaviour-to-Improve-Clinical-Practice-and-Policy

Moschino, L., Zivanovic, S., Hartley, C., Trevisanuto, D., Baraldi, E., & Roehr, C. C. (2020). Caffeine in preterm infants: where are we in 2020? *ERJ Open Research*, *6*(1). https://doi.org/10.1183/23120541.00330-2019

Pearson, N., Naylor, P.-J., Ashe, M. C., Fernandez, M., Yoong, S. L., & Wolfenden, L. (2020). Guidance for conducting feasibility and pilot studies for implementation trials. *Pilot and Feasibility Studies*, *6*(1), 167. https://doi.org/10.1186/s40814-020-00634-w

Steven Conlon, Juliann M. Di Fiore, Richard J. Martin. (2021) Are we over-treating hypoxic spells in preterm infants? Seminars in Fetal and Neonatal Medicine Jun;26(3):101227. doi: 10.1016/j.siny.2021.101227

Tuti, T., Bitok, M., Malla, L., Paton, C., Muinga, N., Gathara, D., Gachau, S., Mbevi, G., Nyachiro, W., Ogero, M., Julius, T., Irimu, G., & English, M. (2016). Improving documentation of clinical care within a clinical information network: an essential initial step in efforts to understand and improve care in Kenyan hospitals. *BMJ Global Health*, *1*(1), e000028. https://doi.org/10.1136/bmjgh-2016-000028

World Health Organization. (2018). *Improving the quality of paediatric care: an operational guide for facility-based audit and review of paediatric mortality.* World Health Organization. https://apps.who.int/iris/bitstream/handle/10665/279755/9789241515184-eng.pdf

World Health Organization. (2020). *Standards for improving the quality of care for small and sick newborns in health facilities.* {World Health Organization}. https://apps.who.int/iris/bitstream/handle/10665/334126/9789240010765-eng.pdf

Zhang, C.-Y., Liu, D.-J., Hua, S.-D., Guo, S., Li, X.-Y., Zhang, B., & An, L.-H. (2020). Caffeine versus aminophylline in combination with oxygen therapy for apnea of prematurity: A

retrospective cohort study. *Experimental and Therapeutic Medicine*, *20*(5), 46. https://doi.org/10.3892/etm.2020.9175

13 APPENDICES

13.1 Appendix I. Schedule of Study Procedures and Evaluations

	STUDY PERIOD						
	Enrolment	Phase I	Phase II				Close-out
TIMEPOINT	*0*	Formative Research	*PDSA 1*	*PDSA 2*	*PDSA 3*	*PDSA 4*	
**ENROLLMENT AND DATA COLLECTION**	X						
Informed consent	X						
Data collection		X	X	X	X	X	
Qualitative assessments, including in-depth interviews, focus group discussions and/or direct observations		X				X	
Neonate monitoring and chart reviews		X	X	X	X	X	
**PROCESSES**							
Develop apnea of prematurity clinical care bundle		X					
Identify barriers to and facilitators for managing apnea of prematurity		X	X	X	X		
Stakeholder engagement		X	X	X	X	X	
Introduce apnea of prematurity clinical care bundle			X				
Modify and optimize apnea of				X	X	X	

prematurity clinical care bundle							
**ASSESSMENTS**							
Analyze formative research data		X					
Analyze quality improvement data			X	X	X	X	X
Monitor impact of quality improvement (adoptability, feasibility, usability, acceptability, adherence, and accessibility)			X	X	X	X	X
OUTCOMES							
Apnea of prematurity clinical care bundle							X
Consider further implementation research recommendations							X

KENYATTA NATIONAL HOSPITAL
APPROVED
25 MAY 2023
KNH/UoN-ERC
P.O. Box 20723 - 00202 NAIROBI

13.2 Appendix II. Informed Consent Forms

Caffeine Feasibility Study for Apnea of Prematurity at Kenyatta National Hospital

Caregiver and Neonate Participant Information and Informed Consent Form for Enrollment

Version 1.2, 09 February 2023

INVESTIGATORS

**Dr. Grace Irimu**<br>Kenyatta National Hospital<br>Hospital Road<br>P O Box: 20723, Nairobi<br>Phone: +254-72-256-4600	**Dr. Mary Waiyego**<br>Kenyatta National Hospital<br>Hospital Road<br>P O Box: 20723, Nairobi<br>Phone: +254-72-161-2393	**Dr. William Macharia**<br>Aga Khan University<br>3rd Parklands Avenue,<br>P O Box 30270, Nairobi<br>Phone: +254-20-366-1017

Part 1. Information Sheet

Introduction

You are being asked for you and your baby to take part in this study because your baby was born early and has been admitted to Kenyatta National Hospital. This study is sponsored by Aga Khan University and is funded by the Bill & Melinda Gates Foundation, an organization that promotes children's health and helps improve healthcare worldwide. The person in charge of this study at this hospital is Dr. Mary Waiyego.

This is a consent form that gives you information about the study and what will happen if you agree for you and your baby to be in the study. You are free to ask questions about the study at any time. If you agree to take part in this study, you will be asked to sign this consent form or make your mark/thumbprint in front of a witness. You will be given a copy of this form to keep. Another copy will stay with the study records.

Your and your baby's participation are completely voluntary. You have the right to decline to participate in this study or withdraw from this study at any time without negative consequences to you or your baby. You and your baby will receive the same care whether you participate in the study or not. Before you decide, you can talk to anyone you feel comfortable with about the research. If there is anything that you do not understand or you are concerned about the study, please ask the study staff at any time.

Why is this study being done?

In Kenya, there is a need to improve the health of babies born too early (premature). The goal of this study is to find the best way to provide safe care for babies like yours which can lead to improved clinical outcomes.

It is also important for us to understand what the parent/ caregiver thinks about the care options offered to your baby in a hospital setting. We may ask to interview you and/or for you to join a group discussion with other parents. By speaking with parents like you, we aim to learn more about the parent/ caregiver experience with and opinions about the care you and your baby have received.

KENYATTA NATIONAL HOSPITAL
APPROVED
25 MAY 2023
KNH/UoN-ERC
P.O. Box 20723 - 00202, NAIROBI

What do we expect to learn from this study?
From this study we expect to learn how to take better care of small babies who stop breathing at this hospital.

What do I have to do if I take part in this study?
If you agree for you and your baby to be in the study, your baby will continue to receive the usual care the hospital provides. In addition, study nurses will observe and collect data from your baby's medical records. We may also invite you to discuss your experience with and opinions about the care you and your baby have received. However, no study activities or data collection will occur until you agree to participate in the study. Only after you have read (or have read to you), discuss, and sign or make a mark/thumbprint on this form, will you and your baby be enrolled in this study.

Upon enrollment, a monitor may be placed on your baby to collect information on your baby's vital signs. The monitors are safe, already used in within the newborn unit for routine care, and do not pose any significant risk or harm to your baby. During your baby's time in the study, your baby will continue to receive the care recommended by the doctors without interruption.

At enrollment, you will be asked to do the following things if you decide you want your baby to be in the study:

- Answer questions about you, your baby, and your family.
- Tell the study staff about any medical problems you and your baby have had.
- Allow your baby's vital signs to be monitored.
- Allow documentation of clinical activities that take place while your baby is in the study (like medication or other treatments).
- Allow the study team to collect information about your baby from other hospital information sources.

In addition, during the study we may approach you and ask to interview you and/or for you to join a group discussion with other parents that will take about 45 to 90 minutes. If you agree to take part in an interview and/or group discussion, you will sit with a study staff member or in a group and discuss your experience participating in the research study. The study staff member might make an audio-recording of the interview. Again, no study activities will begin before they have been fully explained to you, you have let us know that you understand the study, and you have agreed to participate.

What are the alternatives to study participation?
You have the option to not participate in this study. There will be no negative consequences to you or your baby if you choose not to participate in this study. Should you choose not to participate, your baby will continue to receive the usual standard of care from the hospital staff.

You may choose to have your baby participate in the study but decline to participate in an interview or group discussion. You do not have to participate in an interview or group discussion to have your baby continue in the study.

Why would the study staff withdraw my baby from this study early?
The study doctor may need to take your baby out of the study early if:

- The study is stopped by the sponsor, funder, ethics committee, study protocol team or any other regulatory body.
- Your baby is discharged from the hospital or transfered.

KENYATTA NATIONAL HOSPITAL
APPROVED
25 MAY 2023
KNH/UoN-ERC
P.O. Box 20723 - 00202 NAIROBI

- There are other reasons that may prevent you or your baby from completing the study successfully.

What are the risks of being in this study?
Answering some questions may make you feel uncomfortable. You are free to skip any questions that you do not want to answer. Some of the monitors used in the study may cause mild skin irritation which will be avoided by ensuring frequent changes of placement sites, close monitoring and reporting of any reactions.
Are there benefits to taking part in this study?
You and your baby will not receive any additional benefit from being in this study. This study is designed to help better understand how to provide safe and efficient care for premature newborn babies.

What about confidentiality?
All possible measures will be taken to keep your and your baby's personal information confidential. If this study is published, your and your baby's names will not be used, and you and your baby will not be personally identified. Any photographs or videos of your baby will not include your baby's face or identification.

In order to make sure this study is being done properly, your records may be reviewed by:
- Study staff and monitors
- Ethics committees and/or instituitional review boards

You and your baby's study records will be kept at the hospital for at least five years after the study is completed or for the duration required by Kenyan law. If you want the results of the study, let the study staff know that you would like them. If you decide to leave the study, information already collected from your baby will still be used for the study.

What are the costs to me?
There is no cost to you for your and your baby's participation in this study.

Will I receive payment?
You will not receive any payment for your and your baby's participation in this study.

What happens if my baby is injured during the study?
It is unlikely that you or your baby will be injured as a result of being in this study. If you have any concerns related to this study, please immediately contact Dr. Mary Waiyego, our study neonatologist, at 072-161-2393 and she will tell you where your baby can receive treatment.

What are my and my baby's rights as study participants?
Taking part or having your baby take part in this study is completely up to you. You may choose to withdraw you or your baby from study participation at any time. There will be no penalty or loss of benefits to which you and your baby are otherwise entitled. You and your baby will be treated the same no matter what you decide. If you choose to not have you or your baby be in the study, you and your baby will not lose the benefit of services to which you would normally have at this hospital.

We will tell you about new information from this or other studies that may affect you and your baby's health, welfare, or willingness to stay in this study. If you want the results of the study, let the study staff know that you would like them.

A description of this clinical trial will be available on http://www.ClinicalTrials.gov, as required by United States of America (USA) law. This website will not include information that could identify you and your baby. You can search this website at any time.

The research study was reviewed and approved by the Aga Khan University Research Ethics Committee, the Kenyatta National Hospital-University of Nairobi Ethics Research Committee, and the National Council for Science and Technology-National Bioethics Committee.

What do I do if I have problems or questions?
For questions, concerns, or complaints about the study, you or your baby's rights as study participants, or if your baby has a research-related injury, you should contact:

- Dr. Mary Waiyego, Kenyatta National Hospital, Hospital Rd, Nairobi. Tel: 072-161-2393; email: waiyegomary99@gmail.com
- Secretary/Chairperson, Kenyatta National Hospital-University of Nairobi Ethics and Research Committee Telephone No. 2726300 Ext. 44102 email uonknh_erc@uonbi.ac.ke.

KENYATTA NATIONAL HOSPITAL
APPROVED
25 MAY [illegible]
KNH/UoN-ERC
P.O. Box 20723 - 00202, NAIROBI

KENYATTA NATIONAL HOSPITAL
APPROVED
25 MAY 2023
KNH-UoN-ERC
P. O. Box 20723-00202 NAIROBI

Caregiver and Neonate Participant Information and Informed Consent Form for Enrollment

Part 2. Statement of Non-disclosure and Consent

Participant's statement

I have read this consent form or had the information read to me. I have had the chance to discuss this research study with the study staff. I have had my questions answered in a language that I understand. The risks and benefits have been explained to me. I understand that my participation in this study is voluntary and that I may choose to withdraw any time. I freely agree to participate in this research study. I understand that all efforts will be made to keep information regarding my personal identity confidential. By signing this consent form, I have not given up any of the legal rights that I have as a participant in a research study.

In addition, I agree to maintain the confidentiality of other study participant information disclosed during any group discussions or anything observed during my participation in study activities, and to hold in confidence any and all study participant proceedings observed directly or indirectly.

I agree to participate in this research study **Yes** **No**

Participant printed name

____

Participant signature / Thumb stamp

____

Date ______________

Researcher's statement

I, the undersigned, have fully explained the relevant details of this research study to the participant named above and believe that the participant has understood and has willingly and freely given their consent.

Researcher's printed name ____________________ **Date** ____________

Signature ____________________________

Role in the study ____________________

Witness Printed Name (If witness is necessary, a witness is a person mutually acceptable to both the study staff and the participant)

Name _______________________ **Contact information** ______________

Signature /Thumb stamp ______________________ **Date** _____________

Caffeine Feasibility Study for Apnea of Prematurity at Kenyatta National Hospital

Maelezo ya mshiriki kwa mlezi na mtoto mchanga na fomu ya idhini/makubaliano kwa usajili

KENYATTA NATIONAL HOSPITAL APPROVED 25 MAY 2023 P.O. Box 20723-00202, NAIROBI KNH/UON-ERC

Toleo 1.1, 09 Februari 2022

WATAFITI

**Dr. Grace Irimu**<br>Kenyatta National Hospital<br>Hospital Road<br>P O Box: 20723, Nairobi<br>Phone: +254-72-256-4600	**Dr. Mary Waiyego**<br>Kenyatta National Hospital<br>Hospital Road<br>P O Box: 20723, Nairobi<br>Phone: +254-72-161-2393	**Dr. William Macharia**<br>Aga Khan University<br>3rd Parklands Avenue,<br>P O Box 30270, Nairobi<br>Phone: +254-20-366-1017

Sehemu ya 1. Karatasi ya maelezo

Utangulizi
Tunakuomba wewe na mtoto wako mushiriki kwenye utafiti huu kwa sababu mtoto wako alizaliwa mapema na amelazwa hapa hospitali ya Kitaifa ya Kenyatta. Utafiti huu unafadhiliwa ni Chuo Kikuu cha Aga Khan na Wakfu wa Bill & Melinda Gates, shirika ambalo linakuza afya za watoto na kusaidia katika kuboresha huduma za afya duniani kote. Mtu anayesimamia utafiti huu katika hospitali hii ni Dakrari Mary Waiyego.

Hii ni fomu ya idhini ambayo inakupatia maelezo kuhusu utafiti huu na kile ambacho kitafanyika kama utakubali wewe na mtoto wako kujiunga na utafiti huu. Uko huru kuuliza maswali kuhusu utafiti huu wakati wowote. Ukikubali kushiriki kwenye utafiti huu, utaombwa uweke sahihi yako kwenye fomu hii ya idhini/makubaliano ama uweke alama ya kidole gumba kwenye fomu mbele ya shahidi. Utapewa kopi moja ya fomu hii uweke. Kopi nyengine itabaki kwenye kumbukumbu za utafiti.

Kushiriki kwako na mtoto wako kwenye utafiti huu ni hiari kabisa. Uko na uhuru wa kukataa kushiriki au kujitoa kwenye utafiti huu wakati wowote bila ya wewe au mtoto wako kupata matokeo yoyote mabaya. Wewe na mtoto wako mutapata huduma sawa bila kujali iwapo mutashiriki kwenye utafiti huu au la. Kama kuna kitu chochote ambacho hukielewi kuhusu utafiti huu, tafadhali uliza wafanyikazi wa utafiti huu wakati wowote.

Ni kwa nini utafiti huu unafanywa?
Hapa inchini Kenya ipo haja ya kuboresha afya ya watoto waliozaliwa mapema (watoto waliozaliwa kabla ya wakati wao kufika). Lengo la utafiti huu ni kutafuta njia bora zaidi za kutoa huduma salama kwa watoto wachanga kama huyu wako ambazo zinaweza kuleta matokeo bora ya matibabu.
Pia ni muhimu kwetu kuelewa ni kitu gani ambacho mzazi/mlezi anafikiria kuhusu chaguzi/aina za utunzaji zinazotolewa kwa mtoto wako katika mipangilio ya hospitali. Tunaweza kukuomba tufanye mahojiano na wewe/au ushiriki kwenye majadiliano ya kikundi na wazazi wengine. Lengo la kuongea na wazaii kama wewe ni kujua zaidi kuhusu uzoefu wa mzazi/mlezi na mawazo yao kuhusu huduma ambayo wewe na mtoto wako mumepata.
Ni kitu gani ambacho tunatarajia kujifunza kutokana na huu utafiti?

KENYATTA NATIONAL HOSPITAL
APPROVED
25 MAY 2023

Kutokana na utafiti huu tunatarajia kujifunza kuhusu jinsi ya kukuza njia salama na bora ili kutoa huduma zenye ubora wa hali ya juu kwa watoto wanaoacha kupumua katika hospitali hii.

Ni kitu gani nitafanya nikishiriki kwenye utafiti huu?
Ukikubali wewe na mtoto wako mushiriki kwenye utafiti huu, mtoto wako ataendelea kuhudumia kama kawaida. Kwa ziada, wafanyikazi wa utafiti huu wataangalia mtoto wako na recodi zake. Harakati za utafiti huu zitajumuisha kuangalia hali ya mtoto wako na kuuliza maswali kuhusu wewe na mtoto wako. Hakuna harakati za utafiti zitakazofanyiwa mtoto wako kabla hazijaelezwa kwako kikamilifu, utatuambia kama unazielewa harakati za utafiti huu, na umeweka sahihi ama kuweka alama ya kidole gumba katika fomu hii ya makubaliano/idhini. Wewe na mtoto wako mutasajiliwa kwenye utafiti huu tu baada ya kusoma (ama kusomewa), kujadili na kuweka sahihi au alama ya kidole gumba kwenye fomu hii.

Baada ya kusajiliwa kifaa cha kufuatilia kinaweza kuwekwa kwa mtoto wako ili kinakili maelezo kuhusu ishara muhimu za mtoto wako. Vifaa vya kufuatilia viko salalma, tayari vinatumika hapa hospitalini, na haviwezi kusababisha hatari kubwa au madhara kwa mtoto wako. Vifaa vya kufuatilia vinaweza kubaki kwa mtoto wako kwa muda wote ambao mtoto wako atakuwa hospitalini lakini havitaleta usumbufu wowote kwa matibabu ya kawaida kwa mtoto wako. Mtoto wako ataendelea kupata huduma iliyopendekezwa kutoka kwa wahudumu wa hospitali bila usumbufu wowote kwa muda wote ambao atakuwa kwenye utafiti huu.

Wakati wa kusajiliwa utaombwa kufanya mambo yafuatayo ukiamua kwamba mtoto wako ajiunge na utafiti huu:

- Ujibu maswali kuhusu wewe mwenyewe, mtoto wako na familia yako.
- Uwaambie wafanyikazi kwenye utafiti huu kuhusu matatizo yote ya kiafya ambayo wewe na mtoto wako mumekuwa nayo.
- Kuruhusu ishara muhimu za mtoto wako kufuatiliwa.
- Kuruhusu kunakiliwa kwa shughuli zote za matibabu ambazo zitafanyika wakati mtoto wako yuko kwenye utafiti huu (kama vile madawa au matibabu mengine)
- Kuruhusu wafanyikazi kwenye utafiti huu kuchukua maelezo kuhusu mtoto wako kutoka kwenye vyanzo vingine vya maelezo vya hospitali.

Kwa kuongezea, wakati utafiti unapoendelea unaweza kuombwa ufanyiwe mahojiano/ama ushiriki kwenye mahojiano ya kikundi na wazazi wengine ambayo yatachukua kati ya dakika 45 hadi 90. Ukikubali kushiriki kwenye mahojiano/ama kwenye majadiliano ya kikundi, utakaa na mfanyikazi wa utafiti huu/ama utakaa kwenye kikundi ili mujadili kuhusu munayoyopitia/muliyoyapitia kwenye utafiti huu. Mfanyi kazi kwenye utafiti huu anaweza kurekodi sauti zenu wakati wa majadiliano. Pia, hakuna shughuli za utafiti zitakazoanza kabla hazijafafanuliwa kwako kikamilifu, utatuambia iwapo umeuelewa utafiti huu na umekubali kushiriki.

Ni njia gani mbadala za kushiriki kkwenye utafiti huu?
Uko na chaguo la kutoshiriki kwenye utafiti huu. Hakutakuwa na matukio yoyote kwako wewe au kwa mtoto wako ukichagua kutoshiriki kwenye utafiti huu. Ukichagua kutoshiriki kwenye utafiti huu, mtoto wako ataendelea kupata matibabu ya kiwango cha kawaida kutoka kwa wafanyikazi wa hospitali.

Unaweza kuchagua kwamba mtoto wako ashiriki kwenye utafiti huu lakini ukatae kushiriki kwenye mahojiano ama majadiliano ya kikundi. Sio lazima ushiriki kwenye mahojiano ama kwenye majadiliano ya kikundi ndio mtoto wako ashiriki kwenye utafiti huu

Ni kwa nini wafanyikazi wa utafiti wanaweza kumtoa mtoto wangu kwenye utafiti mapema?
Daktari wa utafiti huenda akhitajika kumtoa mtoto wako kwenye utafiti huu mapema iwapo/kama:

- Kama utafiti utasimamishwa ni mfadhili, mwanzilishi, kamati ya kuangalia maadili mema ya utafiti ama chombo chengine cha udhibiti.
- Kama mtoto wako atatolewa hospitalini'
- Kama kutakuwa na sababu zingine ambazo huenda zitakuzuia wewe au mtoto wako kukamilisha utafiti huu kwa mafanikio

Kuna athari gani zinazotokana na kushiriki kwenye utafiti huu?
Kwa kuyajibu maswali mengine huenda ukahisi/ukajisikia una wasiwasi ama kuwa na usumbufu. Uko huru kuruka maswali yoyote ambayo hutaki kuyajibu. Baadhi ya vifaa vya kufuatilia vitakavyotumika huenda vikawasha kidogo kwa ngozi ambayo itaepukwa kwa kuhakikisha mabadiliko ya mara kwa mara kwa maeneo ya uwekaji, ufuatiliaji wa karibu na kuripoti athari yoyote.

Je, kuna manufaa yoyote ya kushiriki kwenye utafiti huu?
Wewe na mtoto wako hamtapata manufaa yoyote ya ziada kwa kushiriki kwenye utafiti huu. Utafiti huu umeundwa ili kusaidia kuelewa zaidi jinsi ya kutoa zilizo salama na zenye utanisi kwa watoto waliozaliwa mapema kabla ya wakati wao kufika.

KENYATTA NATIONAL HOSPITAL APPROVED 25 MAY 2023 BOX 20723 - 00202, NAIROBI

Je, ni vipi kuhusu usiri?
Hatua zote zinazowezekana zitachukuliwa ili kuweka siri maelezo ya kibinafsi yako na ya mtoto wako. Ikiwa utafiti huu utachapishwa, jina lako na la mtoto wako hayatatumika, na wewe pia mtoto wako binafsi hamtatambuliwa.

Ili kuhakikisha kwamba utafiti huu unafanyika vizuri, rekodi zako huenda zikakaguliwa ni:

- Wafanyikazi wa utafiti na wachunguzi,
- Kamati ya kuangalia maadili mema ya utafiti/ama bodi za ukaguzi wa kitaasisi

Rekodi zako na mtoto wako za utafiti zitawekwa hapa hospitalini kwa muda usiopungua miaka mitano baada ya utafiti kukamilika ama kwa muda unaohitajika kisheria ya Kenya. Kama utahitaji majibu ya utafiti huu, mjulishe mfanyikazi wa utafiti. Ukiamua kujitoa kwenye utafiti huu, maelezo ambayo yatakuwa yamepatikana kutoka kwa mtoto wako bado yatatumika kwenye utafiti huu.

Je, kuna gharama gani kwangu?
Hakuna gharama zozote kwako au kwa mtoto wako kwa kushiriki kwenye utafiti huu.

Je, nitapokea malipo yoyote?
Hautapokea malipo yoyote kwa wewe au mtoto wako kushiriki kwenye utafiti huu.

Ni nini kitakachotokea ikiwa mtoto wangu atajeruhiwa wakati wa utafiti?
Haiwezekani kwamba wewe au mtoto wako mutapata majeraha kwa kushiriki kwenye utafiti huu. Ukipata wasiwasi kwa sababu ya kushiriki kwenye utafiti huu, tafadhali wasiliana kwa haraka sana na Daktari Mary Waiyego kupitia numbari ya simu 072-1612393 na atakwambia ni wapi ambako mtoto wako anaweza kupata huduma.

Je, haki zangu na za mtoto wangu ni zipi kama mshiriki wa utafiti?

Wewe ama mtoto wako kushiriki kwenye utafiti huu ni uamuzi wako wewe mwenyewe. Unaweza kuchagua mwanao kuacha kuendelea na taratibu za utafiti hii wakati wowote. – hautapata adhabu yoyote ama kukosa faida zozote ambazo wewe na mtoto wako munastahili kupata. Wewe na mtoto wako mutachukuliwa tu kama kawaida bila kujali uamuzi wako. Ukichagua wewe na mtoto wako kutoshiriki kwenye utafiti, wewe na mtoto wako hamtapoteza faida za kupata huduma ambazo mtapata kama kwaida katika hospitali hii.

Tutakuambia kuhusu habari mpya kutoka kwa utafiti huu au tafiti zingine ambazo huenda zikaaathiri afya yako na ile ya mtoto wako, ustawi, nia ya kutaka kubaki kwenye utafiti huu. Kama unataka majibu ya utafiti huu, mjulishe mfanyikazi kwenye utafiti huu

Maelezo kuhusu utafiti huu yatapatikana kwenye http://www.ClinicalTrials.gov, kama inavyohitajiwa kwa sharia ya United States of America (USA)
Utafiti huu ulipitiwa na kuthibitishwa ni Kamati ya Maadili Bora ya Utafiti ya Chuo Kikuu cha Aga Khan, na Baraza la Kitaifa la Sayansi na Teknologia- National Bioethics Committee.

Je nitafanya nini nikiwa na matatizo au maswali?

Ukiwa na maswali, wasiwasi, au malalamiko kuhusu utafiti huu, haki zako wewe au mtoto wako kama mshiriki wa utafiti, ama ikiwa mtoto wako ako na jeraha ambalo linahusiana na utafiti huu, unatakiwa kuwasiliana na:

- Daktari Mary Waiyego, Hospitali ya Kitaifa ya Kenyatta, Hospital Road Nairobi. Nambari ya simu 072-161-2393; barua pepe:waiyegomari99@gmail.com
- Mwandishi/mwenyekiti, Kamati ya Maadili ya Utafiti ya Hospitali ya Kitaifa ya Kenyatta-Chuo Kikuu cha Nairobi, nambari ya simu 2726300 Ext. 44102 email: uonknh erc@uoni.ac.ke.

Maelezo ya mshiriki kwa mlezi na mtoto mchanga na fomu ya idhini/makubaliano kwa usajili

Sehemu ya 2. Taarifa ya kutofichua na idhini/makubaliano

Taarifa ya washiriki

Nimesoma au nimesomewa fomu ya idhini/makubaliano. Nimepata nafasi ya kujadili utafiti huu na wafanyikazi wa utafiti. Maswali yangu yamejibiwa katika lugha ambayo naielewa. Nimeelezewa athari na faida za utafiti huu. Ninaelewa kwamba kushiriki kwangu katika utafiti huu ni kwa hiari yangu na pia ninaweza kuchagua kujitoa kwenye utafiti wakati wowote. Nimekubali kwa hiari yangu kushiriki kwenye utafiti huu. Ninaelewa kwamba kila mbinu itafanywa ili kuweka maelezo yote yanayonitambulisha kuwa siri. Kwa kusaini fomu hii ya idhini/makubaliano, sijaacha haki yoyote ya kisheria ambazo niko nazo kama mshiriki wa utafiti.

Kwa kuongezea, niakubali kudumisha usiri wa maelezo yaliyotolewa ni washiriki wengine kwenye utafiti wakati wa majadiliano ya kikundi ama kitu chochote nilichoona wakati nikishiriki kwenye shughuli za utafiti, na kushikilia kwa kujiamini na shughuli zote za utafiti nilizoona moja kwa moja au kwa njia isiyo ya moja kwa moja.

Nina kubali kushiriki kwenye utafiti huu **Ndiyo** **La**

Jina la mshiriki --

Sahihi ya mshiriki/alama ya kidole gumba -----------------------------------

KENYATTA NATIONAL HOSPITAL APPROVED 25 MAY [illegible] P.O. Box 20723 - 00202, NAIROBI

Tarehe-----------------------------------

Taarifa ya mtafiti

Mimi, niliyetia sahihi, nimeeleza kwa kikamilifu mambo yote yanayohitajika kwa utafiti huu kwa mshiriki aliyetajwa hapo juu na ninaamini kwamba mshiriki ameelewa na ametoa idhini/amekubali kwa hiari yake bila kushurutishwa/kushawishiwa

Jina la mtafiti--- **Tarehe**--------------------------

Sahihi --

Jukumu lake kwenye utafiti--

Jina la shahidi (kama shahidi anahitajika, shahidi ni mtu ambaye anakubalika katika pande zote kwa mshiriki na utafiti)

Name ______________________________ **Contact information** ____________________

Jina --- **Maelezo ya mawasiliano** ------------------------------------

Signature /Thumb stamp ______________________________ **Date** __________________

Sahihi/alama ya kidole gumba ---------------------------- **Tarehe** ------------------------

APPROVED 25 MAY [illegible] - 00202

Healthcare Provider/ Administrator Participant Information and Informed Consent Form for Enrollment

Version 1.1, 09 February 2022

INVESTIGATORS

**Dr. Grace Irimu**	**Dr. Mary Waiyego**	**Dr. William Macharia**
Kenyatta National Hospital<br>Hospital Road<br>P O Box: 20723, Nairobi<br>Phone: +254-72-256-4600	Kenyatta National Hospital<br>Hospital Road<br>P O Box: 20723, Nairobi<br>Phone: +254-72-161-2393	Aga Khan University<br>3rd Parklands Avenue,<br>P O Box 30270, Nairobi<br>Phone: +254-20-366-1017

Part 1. Information Sheet

Introduction
You are being asked to take part in this study because you are a healthcare provider or administrator involved in the “Feasibility of management of apnea of prematurity with caffeine at a tertiary health care facility in Kenya. A quality improvement study.” This study is sponsored by Aga Khan University and is funded by the Bill & Melinda Gates Foundation, an organization that promotes children's health and helps improve healthcare worldwide. The person in charge of this study at this hospital is Dr. Mary Waiyego.

This is a consent form that gives you information about the study and what you will have to do if you agree to be in the study. You are free to ask questions about the study at any time. If you agree to take part in this study, you will be asked to sign this consent form. You will be given a copy of this form to keep. Another copy will stay with the study records.

Your participation is completely voluntary. You have the right to refuse to join or withdraw from the study at any time without negative consequences to you or your employment. Before you decide to participate, you can talk to anyone you feel comfortable with about the research. If there is anything that you do not understand about the study, please ask the study staff or Dr. Waiyego at any time.

Why is this study being done?
In Kenya, there is a need to improve the health and survival of premature babies. A serious health concern, apnea of prematurity is when premature babies experience difficulty breathing, including stopping breathing for short periods of time. The goal of this study is to find the best way to provide safe, effective, and high-quality care which will lead to improved clinical outcomes for newborn babies with apnea of prematurity. As part of this study, we aim to learn more about healthcare providers' and administrators' experiences with and perceptions around introducing caffeine citrate as part of a clinical care bundle to manage apnea of prematurity. Healthcare providers and administrators involved in supporting premature neonates at this hospital during the study period may be asked to take part in this study.

What do we expect to learn from this study?

From this study, we expect to learn from healthcare providers and administrators their thoughts, attitudes, and practices around the introduction of caffeine citrate as part of a clinical care bundle to manage apnea of prematurity in this hospital as well as barriers and facilitators.

What do I have to do if I take part in this study?
If you agree to take part in the study, you may participate in one or more study discussions. A study visit may consist of an in-depth interview, participation in a focus group discussion, and/or direct observations. These study visit may last up to 90 minutes. No study activities will be initiated before they have been fully explained to you, you have let us know that you understand the study activities, and you have signed this consent form. Only after you have read, discussed, and signed this form, will you be enrolled in this study. After signing this consent form, you will be enrolled and you will participate in discussions or answer questions about your experience with and perceptions around providing caffeine for apnea of prematurity and the use of monitors. Audio-recordings may be made of the study visits.

What are the alternatives to study participation?
You have the option to not participate in this study. There will be no consequences to you or your employment if you choose not to participate in this study.

What are the risks of being in this study?
Answering some questions may make you feel uncomfortable. You are free to skip any questions that you do not want to answer.

Are there benefits to taking part in this study?
There are no direct benefits to you for taking part in this study. This study is designed to help better understand how to provide safe, efficient, and high-quality care for premature newborn babies in this hospital. This may benefit the clinical processes for hospital staff and clinical outcomes for babies in the future.

What about confidentiality?
All possible measures will be taken to keep your personal information confidential. All personal information during the study will be kept confidential. If this study is published, your name will not be used, and you will not be personally identified. Any photographs or videos will not include your face or identification.

In order to make sure this study is being done properly, your records may be reviewed by:
- Study staff and monitors
- Ethics committees and/or institutional review boards

Your study records will be kept at the hospital for at least five years after the study is completed or for the duration required by Kenyan law. If you want the results of the study, let the study staff know that you would like them. If you decide to leave the study, information already collected from you will still be used for the study.

What are the costs to me?
There is no cost to you for your participation in this study.

Will I receive payment?
You will not receive any payment for your participation in this qualitative study.

What are my rights as a study participant?

Taking part in this study is completely voluntary and you may choose to withdraw your participation at any time. If you choose not to be in the study, there will be no penalty to you or to your employment. We will tell you about new information from this or other similar studies that may affect your willingness to stay in this study. If you want the results of the study, let the study staff know that you would like them.

A description of this clinical trial will be available on http://www.ClinicalTrials.gov, as required by United States of America (USA) law. This website will not include information that could identify you. You can search this website at any time.

The research study was reviewed and approved by the Aga Khan University Research Ethics Committee, the Kenyatta National Hospital-University of Nairobi Ethics Research Committee, and the National Council for Science and Technology-National Bioethics Committee.

What do I do if I have problems or questions?
For questions, concerns, complaints about the study or your rights as a study participant, you should contact:

- Dr. Mary Waiyego, Kenyatta National Hospital, Hospital Rd, Nairobi. Tel: 072-161-2393; email: waiyegomary99@gmail.com
- Secretary/Chairperson, Kenyatta National Hospital-University of Nairobi Ethics and Research Committee Telephone No. 2726300 Ext. 44102 email uonknh_erc@uonbi.ac.ke.

KENYATTA [illegible]
APPROVED
25 MAY [illegible]
[illegible]

Healthcare Provider/ Administrator Participant Information and Informed Consent Form for Enrollment

Part 2. Statement of Non-disclosure and Consent

Participant's statement

I have read this consent form or had the information read to me. I have had the chance to discuss this research study with the study staff. I have had my questions answered in a language that I understand. The risks and benefits have been explained to me. I understand that my participation in this study is voluntary and that I may choose to withdraw any time. I freely agree to participate in this research study. I understand that all efforts will be made to keep information regarding my personal identity confidential.

In addition, I agree to maintain the confidentiality of other study participant information disclosed during any group discussions or anything observed during my participation in study activities, and to hold in confidence any and all study participant proceedings observed directly or indirectly.

By signing this consent form, I have not given up any of the legal rights that I have as a participant in a research study.

I agree to participate in this research study: **Yes** **No**

Participant printed name

____

Participant signature

____________________________________ **Date** ________________

KENYATTA NATIONAL HOSPITAL
APPROVED
25 MAY
P.O. Box

Researcher's statement

I, the undersigned, have fully explained the relevant details of this research study to the participant named above and believe that the participant has understood and has willingly and freely given their consent.

Researcher's printed name ____________________ **Date** ____________

Signature ____________________________

Role in the study ____________________

13.3 Appendix III. Study Data Elements

Participant demographic and medical history/chart information will be collected for neonate study participants. Below are examples of neonatal and maternal data that may be collected:

- Date of birth, gestational/corrected neonatal age
- Sex
- Weight
- Socio-demographic information
- Obstetric history including gravidity and parity, duration of current pregnancy, mode of delivery, Apgar score results
- Comorbidities and medical history since birth, including current medications and treatments
- Neonatal vital signs, number and frequency of apneic episodes (if any)
- Dosing, timing, duration, and changes to aminophylline or caffeine citrate treatment
- Timing and duration of vital signs, desaturations, and clinical condition (if any)
- Clinical monitoring recorded data from clinical monitor (spot check or continuous data collection)
- Apnea-specific information such as time and duration of apneic episodes, cause of apnea, interventions provided

Qualitative feedback investigating adoptability, feasibility, usability, acceptability, adherence, and accessibility will be collected through in-depth interviews and focus group discussions with healthcare personnel and caregivers as well as by observations at the newborn unit. In-depth interviews and focus group discussions will utilize interview and discussion guides, respectively. A checklist will be used for participant observations.

KENYATTA NATIONAL HOSPITAL
APPROVED
25 MAY [illegible]
P.O. Box [illegible] - 00202
